# Fresh and dry fruit production in Himalayan Kashmir, Sub-Himalayan Jammu and Trans-Himalayan Ladakh, India

**DOI:** 10.1016/j.heliyon.2020.e05835

**Published:** 2021-01-11

**Authors:** Rayees Ahmad, Barkat Hussain, Tariq Ahmad

**Affiliations:** a Department of Zoology, University of Kashmir, Srinagar 190006, India; b Division of Entomology, Faculty of Horticulture, SKUAST, Kashmir 190025, India

**Keywords:** Fresh fruit, Dry fruit, Production, Employment, Soil fertility, Ladakh, Jammu & Kashmir

## Abstract

Jammu & Kashmir and Ladakh are two union territories (UT's) famous for their beauty and cool climate. Both the UT's are familiar with their horticulture production at the national and international level. Jammu & Kashmir offers enough scope for cultivation of all types of horticulture crops covering various sub-tropical fruits like mango, guava, citrus, litchi, temperate fruits like apple, pear, peach, plum, apricot, almonds, cherry, and apricot. Horticulture has emerged as a fast-growing sector in Jammu & Kashmir and to some extent in Ladakh, offers a wide range of opportunities to farmers for crop diversification. Horticulture has proved its credibility in improving income through increased productivity, creating employment, and strengthening exports. Jammu & Kashmir comprises 20 districts and Ladakh two districts, and both having comparative advantages in some specific types of fruit cultivation due to variations in soil fertility, soil matter, topography, and variable to harsh weather. In this survey, we analyze the district wise contribution and improvement of horticulture production of fresh fruits and an increase in their growing area. The survey-based on secondary data sources collected from the Directorate of Horticulture of Jammu & Kashmir and Ladakh. The data is analytical and empirical, directly contributing to the horticulture production of India. The area and production of apple are maximum in district Baramulla, a temperate region of J&K. While, maximum area as well as the production of apricot is in district Leh, a high altitude and cold arid region of Ladakh.

## 1 Introduction

The state (Jammu & Kashmir), has been recently divided into two Union territories – Jammu & Kashmir and Ladakh under the Jammu & Kashmir Reorganization Act, 2019. The UT's of Jammu & Kashmir and Ladakh are the two significant zoo-geographical regions located in the Northern part of the Indian-subcontinent around the Karakorum and the Western Himalayan regions. UT's of Jammu & Kashmir and Ladakh are fertile lands, a favorable environment for the plentiful production of both fresh and dry fruits. Fruits are nature's marvelous gift to the human, packed with vitamins, minerals, antioxidants, and other nutrients. Both dry and fresh fruits have therapeutic potential against harmful diseases. Jammu & Kashmir is famous for its marvelous mountains, glaciers, rivers, lakes, wetlands, croplands and, beautiful valleys, and the biomass state of India because of its vast biodiversity and pleasing location. According to census 2011, the total population of Jammu & Kashmir is 12548926 which contributes 1.04% to the total population of India. The area of 20441 km^2^ forms the total forest area and the net area irrigated in J&K state is 313000 ha. Both UT's have international borders with Afghanistan and China in the North and national boundaries with Punjab and Himachal Pradesh towards the South. Seven physiographic zones (Hussain, 2000) of Jammu & Kashmir and Ladakh are closely associated with the structural parts of the western Himalayas and include Plains, Foothills, Lesser Himalayas, Great Himalayas, Valley of Kashmir, Upper Indus valley, and Karakoram regions. Geographically these UT's are divided into 4 distinct zones, sub-mountain and semi-mountain plain known as Kandi or dry belt; the Shivalik ranges; the high mountain zone forming the Kashmir Valley, Pir-Panjal range and its offshoots including Doda, Poonch and Rajouri districts and part of Kathua, and Udhampur districts; the middle run of the Indus river comprising Leh and Kargil. The UT of Ladakh has a remote mountain beauty and its distinct culture. The highest temperature is experienced in June and July and the lowest in January. The Jammu district offers a great variation in the topographic, ecological, and soil management aspects, soil pH varies throughout the district which ranging from 4.67 to 9.08 with a mean value of 7.07. The variability in average pH, Phosphorous, Nitrogen content, and organic matter were found in soils of Kishtwar, Pulwama, Budgam, and Anantnag (Muzaffar et al., 2018). Lime induced chlorosis and alkaline pH and soils high in potassium have been reported as a limiting factor in peach, almond and apricots. These factors are also responsible for least nutrient uptake, behavior of rootstocks, toxicity of plants, growth, fruit quality and quantity, plant canopy, and other physiological factors (Romera et al., 1991; Morris et al., 1980; Daliakopoulos et al., 2016) The climate of both the UT's is different from the rest of the Indian union and divided into six seasons. These variable seasons of Jammu & Kashmir and Ladakh Union are shown in Table 1.

**Table 1:** Showing different seasons in Union Territories of Jammu & Kashmir and Ladakh.

Season	Period	Local name
Spring	16th March – 15th May	Sonth
Summer	16th May – 15th July	Grisham/Ratkol
Rainy	16th July – 15th September	Wahrat
Autumn	16th September – 15th November	Harud
Winter	16th November - 15th January	Wand
Ice cold	16th January – 15th March	Sheshur

## 2 Union Territory of Jammu & Kashmir

Jammu & Kashmir comprises two distinct climatic regions – Himalayan Kashmir (Moderate or Temperate Kashmir Valley) and Sub-Himalayan Jammu (Hot or Humid Subtropical Jammu).

## 3 Himalayan Kashmir

**Figure 1:**
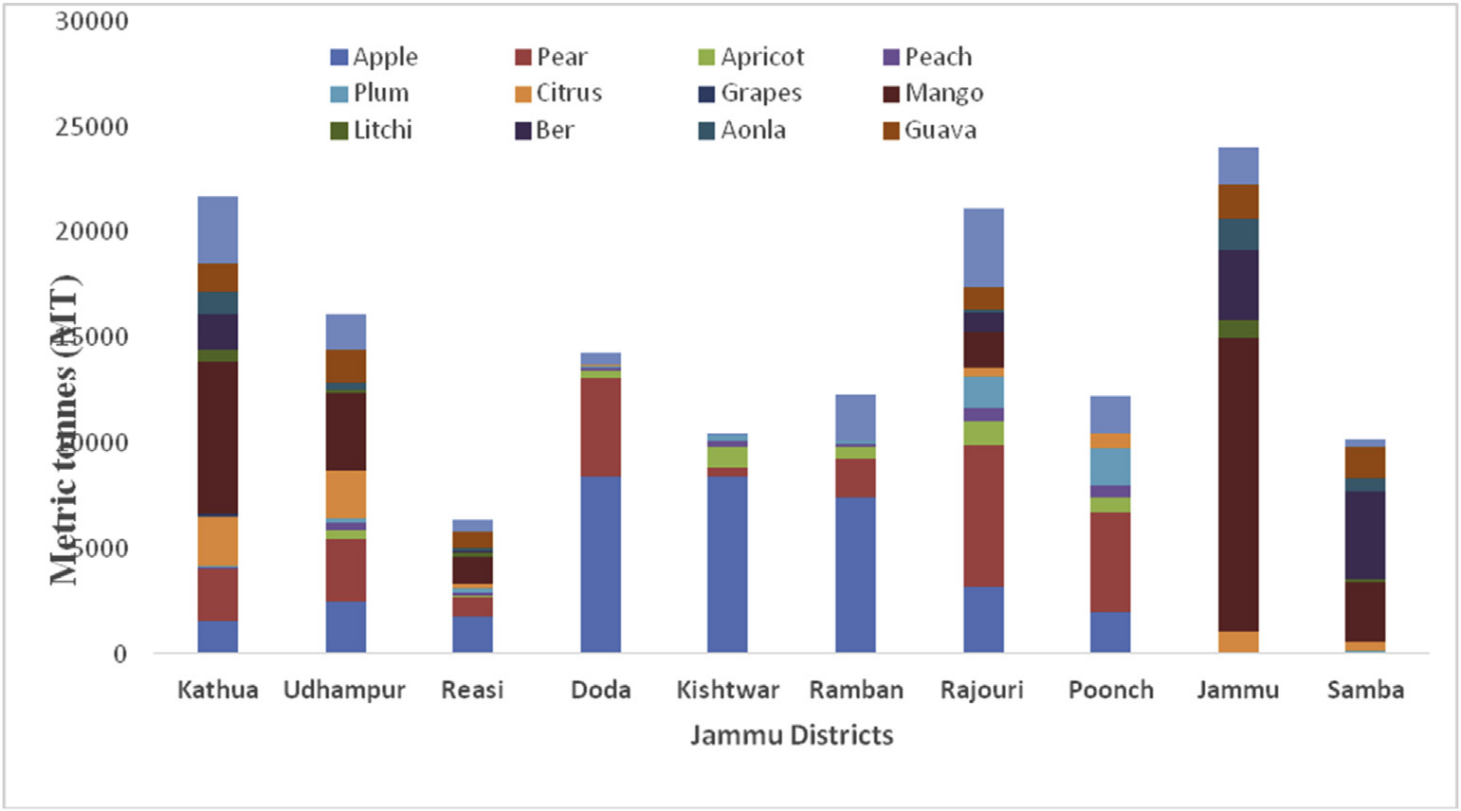
District wise production in metric tons (MT) from the different districts of Jammu region during 2018–2019.

Kashmir valley is the real paradise on Earth, is an intermountain valley bounded on the Southwest by the Pir-Panjal range and on the Northeast by the main Himalayan range. The geographical location of Kashmir valley lies between 33º55ʹ N Latitude and 74º30ʹ East Longitude, and elevation of 1,615m above sea level. Kashmir has a moderate temperature with maximum temperature rises to 32 °C in July and minimum -15 °C in December and January. In both the UT's, Kashmir valley has the most fertile land with a favorable environment for the plentiful production of both fresh and dry fruits. The total area of 210075 ha of land in 2018–19 brought under horticulture cultivation in Kashmir Valley which comprises 10 districts (Table 2). The total cultivated area is 158499 ha (75.45%) under fresh fruits and 51576 (24.55%) remained under dry fruit production, respectively. In 2018–19 the total production of 2145490 MT produced from the Kashmir valley, frames 1940250MT (90.43%) fresh fruits and 205239MT (9.57%) dry fruits, respectively. The main fruits produced in the Kashmir valley are apples locally known as Sebh or Tsoonth, with 1848483MT being produced in the year 2018–19, followed by pear (61154MT), cherry (11747MT), plum (7698MT), apricot (3470MT), peach (2680MT), grapes (750MT), strawberry (425MT) and other fruit crops (3701MT). District Baramulla is one of the leading producers of apples in India, producing 404089MT apples. While as, district Budgam is the largest producer of pear (17685MT), and plum (2719MT). During 2018–19, the maximum production of cherry (3616MT), grapes (606MT), and apricots (757MT) were produced from district Ganderbal. As of 2018–19, Kulgam district is the leading producer of peaches with a production of 412MT. highest production of strawberry (377MT) in 2018–19, produced from district Srinagar of Kashmir valley. The other fresh fruit producing districts in kashmir Valley are presented in Figure 1.

**Table 2:** Locations of all districts of Kashmir valley of Union Territory of Jammu & Kashmir.

S. No.	District	Coordinates	Average elevation	Area
01	Anantnag	33 ^o^73ʹN 75 ^o^15ʹE	1601m	3574 km^2^
02	Bandipora	34º25ʹN 74º39ʹE/34 ^o^42ʹN 74 ^o^65ʹE	1581m	345 km^2^
03	Baramulla	34º11ʹN 74º21ʹE/34 ^o^19ʹN 74 ^o^36ʹE	1593m	3353 km^2^
04	Budgam	34º1ʹN 74º46ʹE/34^o^02ʹN 74^o^87ʹE	1610m	1370 km^2^
05	Ganderbal	34 ^o^23ʹN 74^o^ 78ʹE	1950m	1979 km^2^
06	Kulgam	33º38ʹN 75º01ʹE	1739m	1067 km^2^
07	Kupwara	34º31ʹN 74º15ʹE/34 ^o^52ʹN 74^o^25ʹE	1577m	950 km^2^
08	Pulwama	33º52ʹN 74º53ʹE/33^o^87ʹN 74^o^89ʹE	1630m	1398 km^2^
09	Shopian	33º43ʹN 74º50ʹE/33.72ºN 74.83ºE	2057m	613 km^2^
10	Srinagar	34º05ʹN 74º50ʹE/34^o^08ʹN 74^o^83ʹE	1620m	1979 km^2^

## 4 Sub-Himalayan Jammu

**Figure 2:**
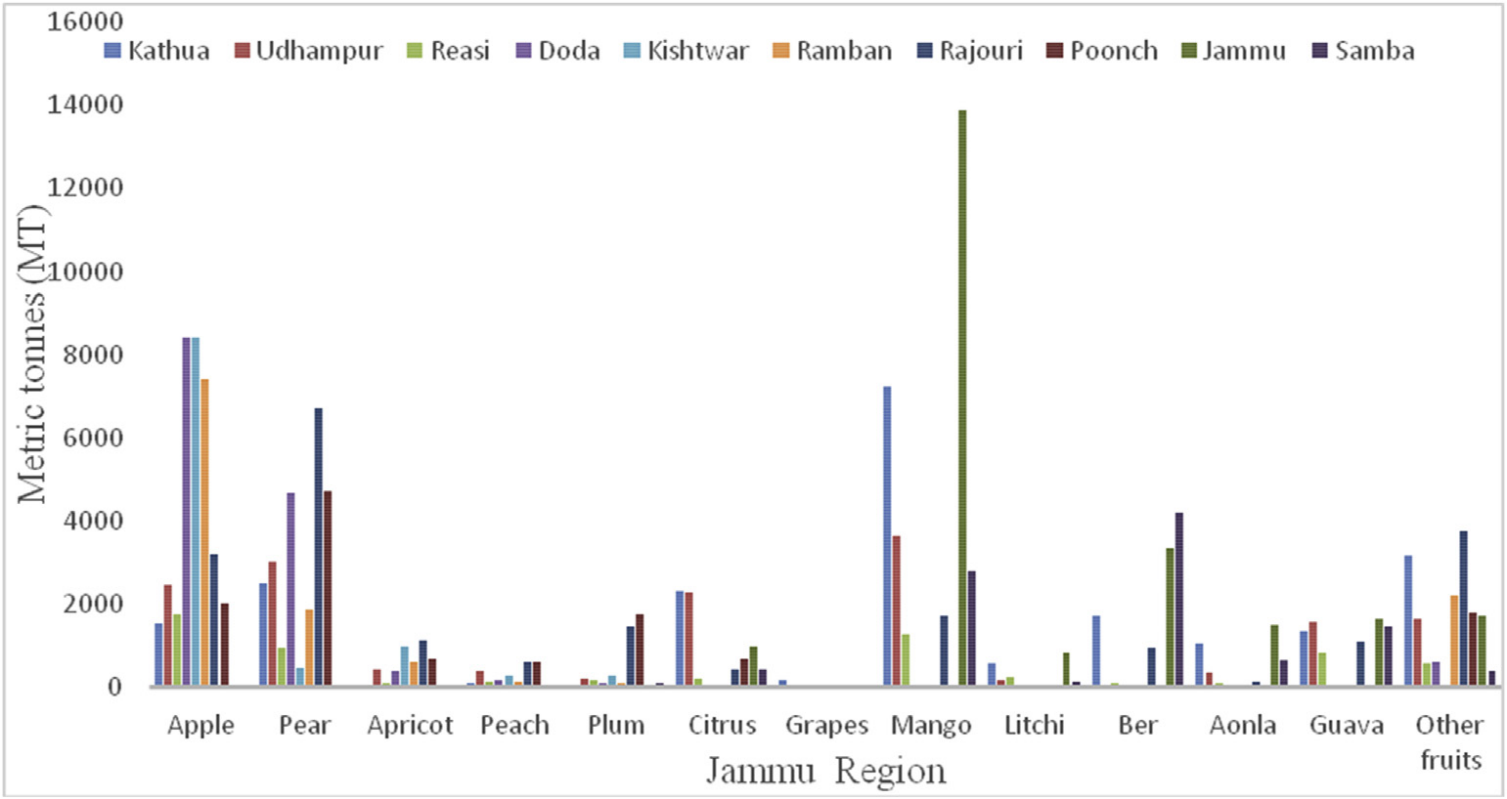
Crop wise production in metric tons (MT) from the different districts of Jammu region during 2018–2019.

**Figure 3:**
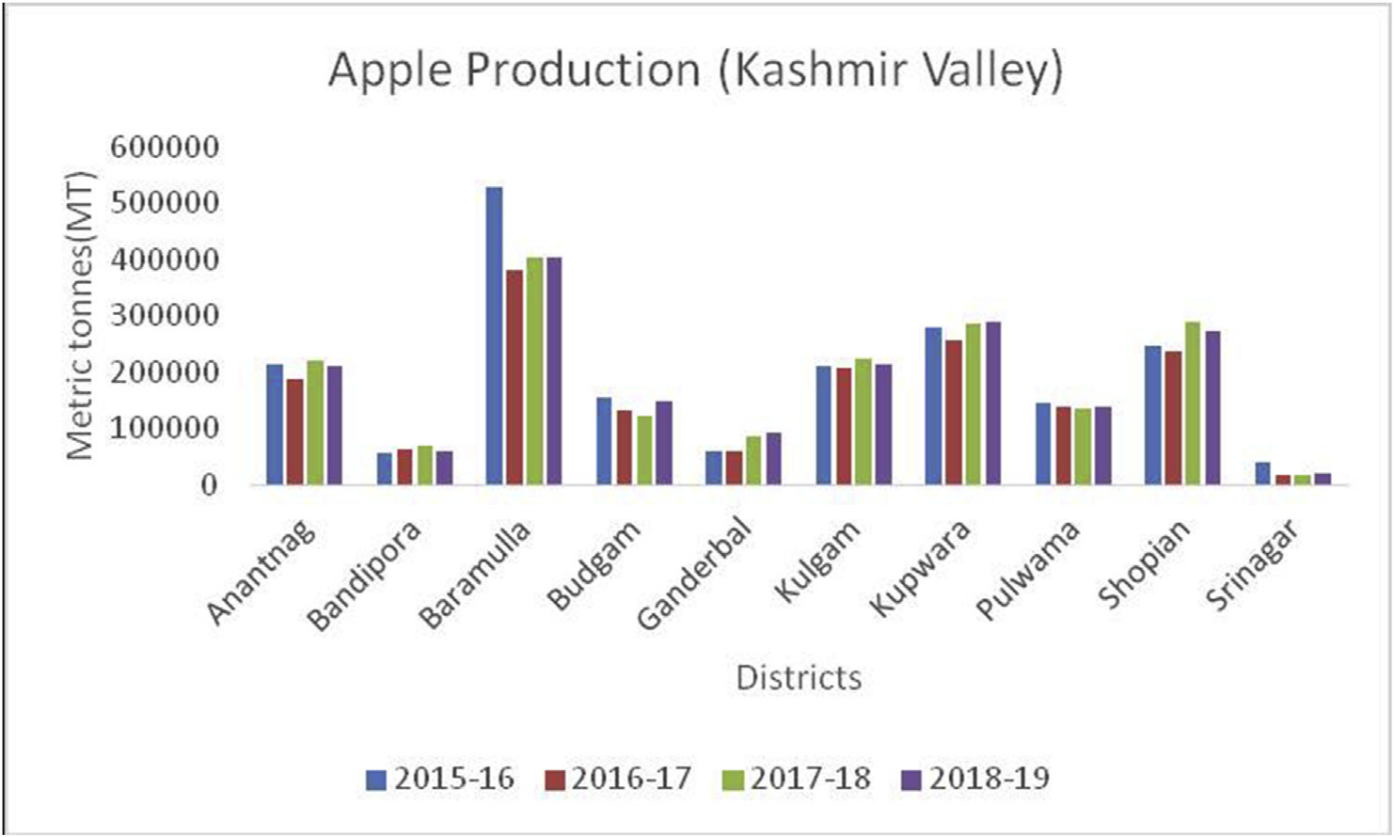
Apple production in metric tons (MT) from 2015-2019 in Kashmir Valley of Union Territory of Jammu & Kashmir.

**Figure 4:**
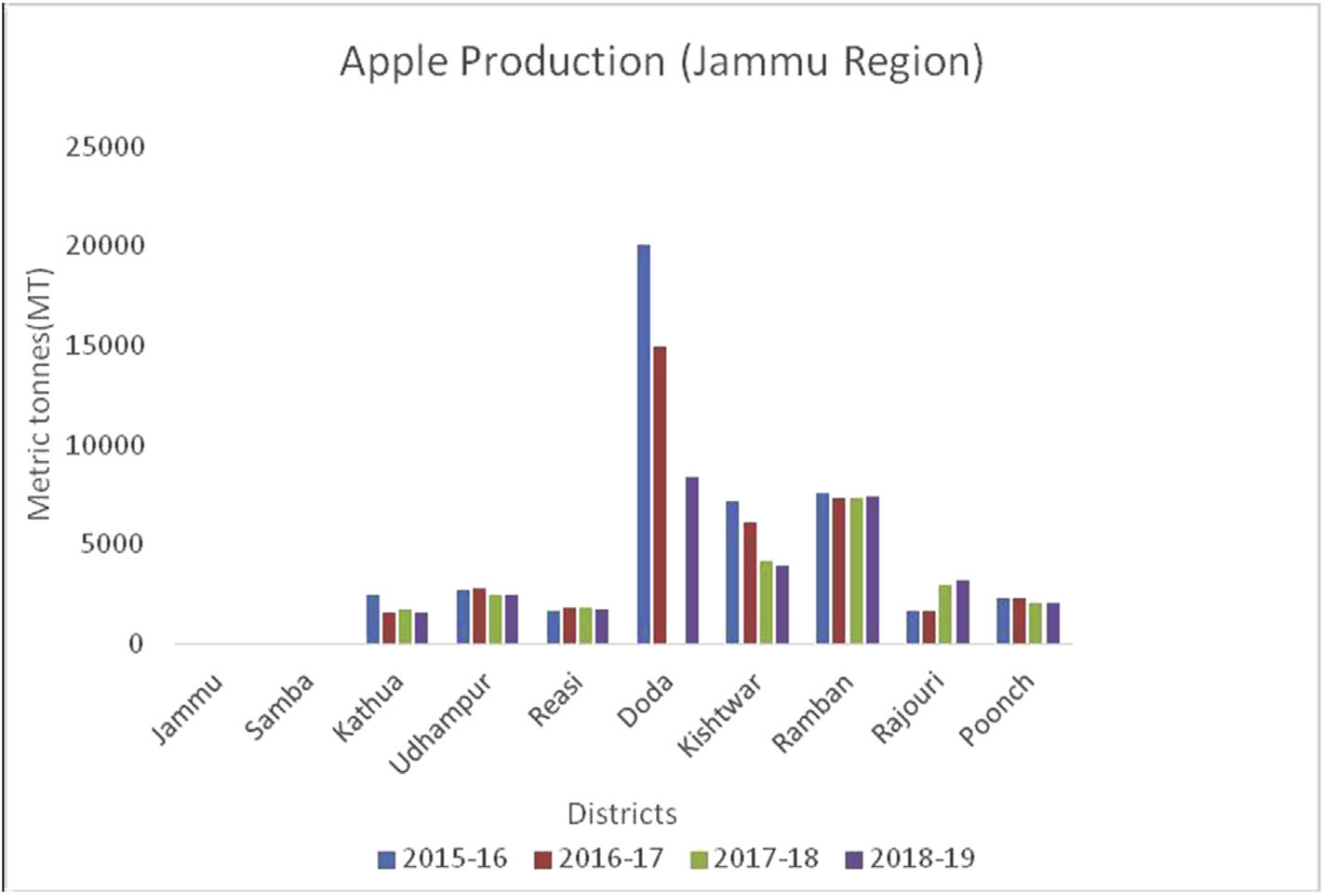
Apple production in metric tons (MT) from 2015-2019 in Jammu region of Union Territory of Jammu & Kashmir.

**Figure 5:**
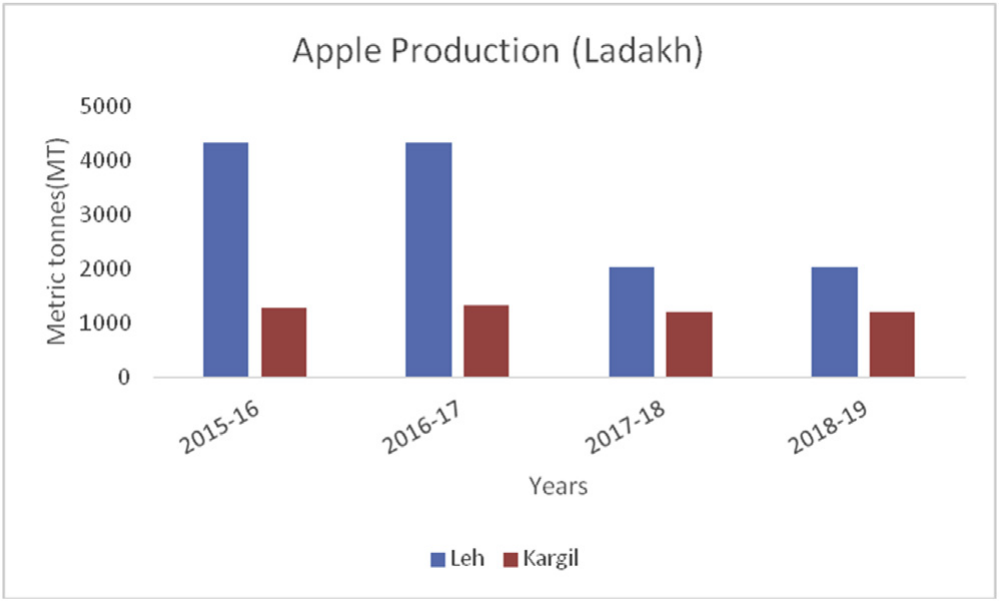
Apple production in metric tons (MT) from 2015–19 from Union Territory of Ladakh.

Sub-Himalayan Jammu lies between32º73ʹ N latitude and 74º87ʹ E longitude and falls in the sub-tropical zone. The total area of the Jammu region is 26293 km^2^ with a total population of 5350811. Pir-Panjal range separates the Jammu from the Kashmir valley. Geographically, the eight sub-regions of Jammu are classified as Shiwaliks, Pir-Panjal belt, Ravi-Tawi Kandi plains, Chenab valley, Gandoh valley, Baderwah valley, Marwan and Marwah valley, and Padder valley. The highest temperature reaches up to 46 °C in June and the lowest 4 °C in January. The maximum precipitation of 42 inches yearly falls from June to September. The soil texture of Jammu varies from loamy sand to sandy loam and is mainly classified under Entisols. Jammu comprises 10 districts, and their locations and average elevations are being shown (Table 3). The maximum area under horticulture cultivation is in district Kathua followed by Rajouri, Jammu, Poonch, Doda, Udhampur, Reasi, Ramban, Samba, and Kishtwar. Maximum district wise production of fresh fruits was recorded from Jammu district followed by Kathua, Rajouri, Udhampur, Doda, Samba, Poonch, Ramban, Reasi, and Kishtwar during 2018–2019 (Figure 2). The main apricot and other fresh fruit producing districts are presented in Figures 3 and 4, respectively. The total area of 118494 ha already under horticulture cultivation in 2018–2019, and from this cultivated area, the total area of 79231 ha under fresh fruits and 39263 ha under dry fruits, respectively. The total production of 253684MT, which constitutes 169251MT (66.72%) for fresh fruits and 84433MT (33.28%) under dry fruits from Jammu region during 2018–2019. During 2018–19, Jammu district tops in the production of mangoes, guava, aonla, and litchi, with a total production of 13876MT, 1649MT, 1506MT, and 847MT, respectively (Figure 5). Kathua district is the leading producer of citrus (2,308MT) and grapes (156MT) 2018–19). Walnut is one the most important dry fruit and its production is shown in the decreasing order from the districts are Doda (21610MT), Poonch (13060MT), Kishtwar (11668MT), Udhampur (9845MT), Ramban (7600MT, Rajouri (7429MT), Kathua (6152MT), Reasi (6992MT). In 2018–19, district Rajouri is the leading producer of peanuts with a total production of 50MT from the Jammu division. District Kathua, leading in the production of almonds with a production of 4MT in the year 2018–19.

**Table 3:** Locations of all districts of Jammu region of Union Territory of Jammu & Kashmir.

S. No.	District	Coordinates	Average elevation	Area
01	Doda	33º08ʹN 75º34ʹE/3313ºN 75^o^57ʹ E	1107m	11691 km^2^
02	Jammu	32º44ʹN 74º52ʹE/32^o^.73ʹN 74^o^87ʹE	350m	3097 km^2^
03	Kathua	32^o^38ʹN 75^o^51ʹE	393m	2651 km^2^
04	Kishtwar	33^o^.32ʹN 75^o^77ʹ E	1638m	7737 km^2^
05	Poonch	33º46ʹN 74º05ʹE/33^o^ 76ʹN74^o^09ʹE	915m	1674 km^2^
06	Rajouri	33º23ʹN 74º18ʹE/33.38° N 74 ^o^3ʹ E	915m	2630 km^2^
07	Ramban	33^o^ 25ʹ N 75 ^o^25ʹE	747m	1329 km^2^
08	Reasi	33 08° N 74.83ºE	466m	1719 km^2^
09	Samba	32 ^o^57ʹN 75 ^o^12ʹE	384m	914 km^2^
10	Udampur	32º54ʹN 75º08ʹE/32 ^o^91ʹN75^o^ 14ʹE	755m	4550 km^2^

## 5 Union Territory of Ladakh

**Figure 6:**
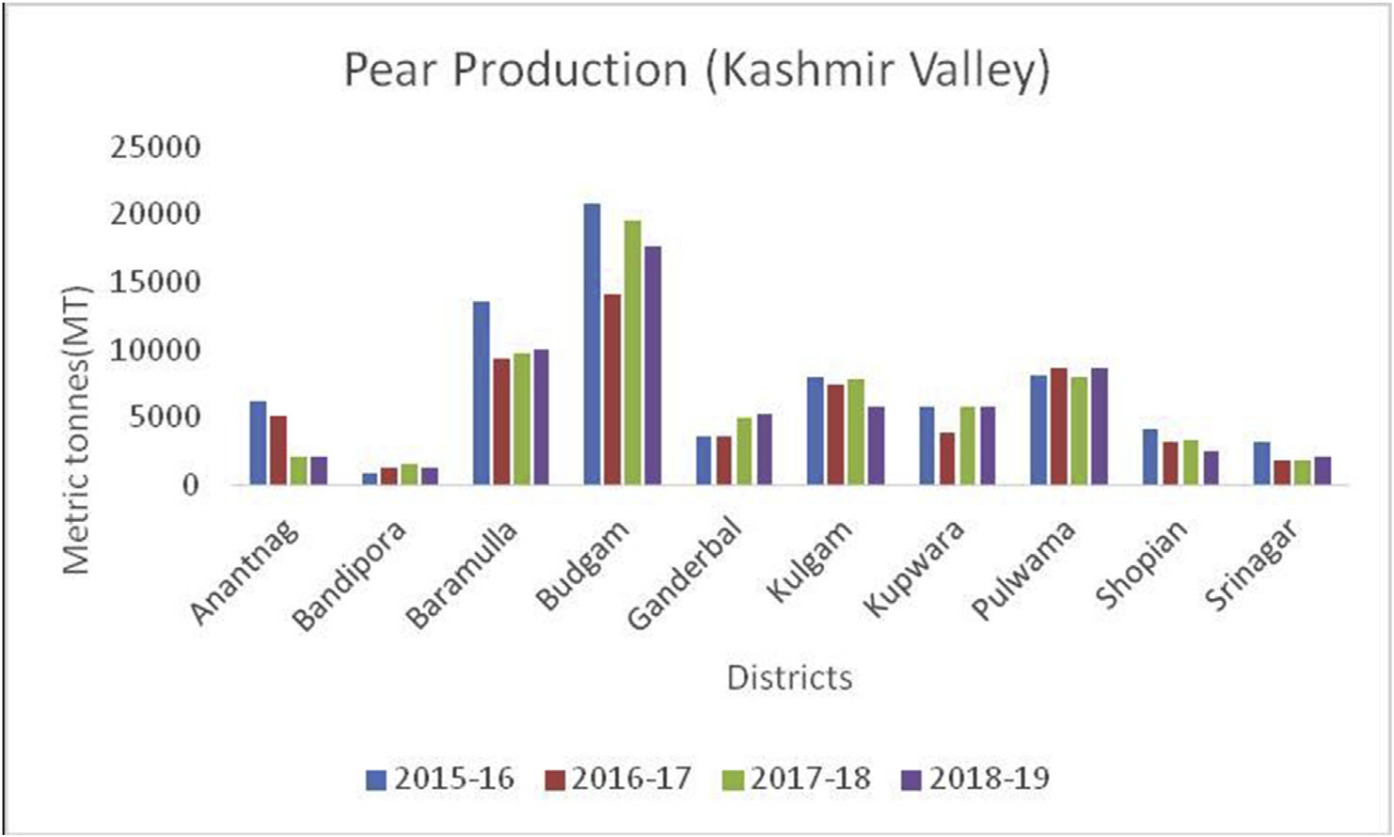
Pear production in metric tons (MT) from 2015-2019 in Kashmir Valley of Union Territory of Jammu & Kashmir.

The Union Territory of Ladakh comprises the Trans-Himalayan region (Cold arid desert). Besides, cold arid climate, it is the highest plateau in the Northern part of India, and the land of high passes with an average elevation of 2,987m. The geographical location lies between 34º10ʹ N Latitude and 77º48ʹ E Longitude which extends from the main Great Himalayas to the Siachen Glacier in the Karakoram Range and includes the upper Indus River valley. Ladakh is famous for its unique culture and remote mountain beauty which is divided into two districts (Table 4). Their Apricots are famous throughout the world due to their high sugar content (Angmo et al., 2017). The total horticultural area is 2,968 ha during 2018–2019 of which 2901 ha (97.74%) are under fresh fruits cultivation and 67 ha (2.26%) under dry fruits cultivation. The fruits produced in the UT of Ladakh are apricots, with 12,686MT, produced in the year 2018–19, followed by apple (3241MT), cherry (43MT), pear (37MT), peach (34MT), grapes (29MT), and plum (12MT). During the crop year 2018–19, Kargil top's in production of apricot, peach, plum, cherry, and grapes with 7776MT, 22MT, 6MT, 31MT, 25MT production respectively. Maximum production of apples (2038MT) and Pear (20MT) reported from district Leh during the year 2018–19 (Figure 6).

**Table 4:** Locations of all districts of Union Territory of Ladakh.

S. No.	District	Coordinates	Average elevation	Area
01	Leh	34º10ʹN 77º40ʹE	3500m	45110 km^2^
02	Kargil	34º33ʹN 76º08ʹE	2676m	14036 km^2^

India is mainly an agricultural country and agriculture is considered the backbone of the Indian economy, even during the Covid-19 pandemic, the growth of this sector shows an upward trend (Goyal, 2020). About 70% of Indian citizens rely on agriculture for their livelihood security. Besides to ensure food security for future generations, the productivity of all such fruits is essential to feed the human population of India which is not still under general equilibrium position. As census report 2011, the total population of farmers of India is 118.7 million and 144.3 million agricultural laborers which consist of 30.55% of the total rural population. Horticulture crops in India contribute about 30% to agricultural GDP. Fresh fruits are consumed by a broad array of consumers, including vegetarians and non-vegetarians, and even domestic and wild animals. The most economically important fresh fruits include apple, pear, mango, apricot, peach, citrus, and litchi, etc. The fruits such as raspberry, blackberry, and gooseberries are a good source of phenols, anthocyanins, and ascorbic acid which play an important role in human health (Moyer et al., 2002; Pantelidis et al., 2007). Besides, strawberries, blackberries, ber and gooseberries have anticancer and anti-proliferative properties (Pillai, 2020) through various modes of action, including induction of metabolizing enzymes, modulation of gene expression, subcellular signaling pathways and programmed cell death (Nile and Park, 2014. Apple is a fresh fruit and growers as well as consumers are highly sensitive towards its quality and taste. India is the second largest producer of fruits and vegetables in the world after China. India is leading in several horticultural crops, namely mango, banana, papaya, cashewnut, arecanut, potato and Ookra. Fruits and vegetables account for 90% of total horticulture production in India.

At the global level, India is at a second position in the production of fruits and vegetables after China and exports different kinds of fresh fruits which includes grapes, which occupy the premier position in exports with 18822.16MT in 2017–18, valued INR 189994.86 Lakhs. Other fresh fruits occupy a great position in global export. Among other fresh fruits, bananas (101314.37MT) valued INR 34877.39 and mango (49180.46MT) valued INR 38234.02. Vietnam is the global leader in cashew nut production (24.9%), followed by Nigeria (19.6%) and India (13.7%) in the year 2016. China is the global leader in potato production (99205600MT), followed by India (48605000MT), respectively during the year 2017–18. The production of horticulture crops during 2017–18, estimated record production of 311.71MT from an area of 25.43 million hectares which is 10.0 % higher than the previous five years. The production of fresh fruits increased from 50.9 million tons to 97.35 million tons from 2004–05 to 2017–2018.

The objectives of this study are (1) to access the present contribution of Union Territories of Jammu & Kashmir and Ladakh for fresh and dry fruits to India and abroad; (2) to analyze the district wise production of fresh fruits in Union territories of Jammu & Kashmir and Ladakh; (3) to make a crop-wise comparison of area and production of horticulture crops for four years; and (4) to give some suggestions for increasing the production of horticulture sector and to increase the income of the growers.

## 6 Materials and methods

The paper is based on secondary data source collected from the Directorate of Horticulture, J&K and Ladakh, economical survey, statistical digest, magazines, and journals which is analytical and empirical. Crop-wise area in hectares, as well as production in metric tons (MT) of dry and fresh fruits for 4 years from 2015–16 to 2019–20, were assessed for easy understanding and interpretation.

## 7 Results and discussion

During 2015–2016, 90.2 million metric tons of fruits were produced by India (National Horticulture database published by National Horticulture Board) in which Kashmir contributes about 27% alone, to the Indian production. In 2015–2016, an area of 337677 ha, forms 71.42% under fresh fruits and 28.58% under dry fruits in J&K. From this area, the production of 2522785MT fruits were produced, including both fresh (88.97%) and dry (11.02%) fruits. Apple constitutes the largest production in UT of Jammu & Kashmir (86.55%) followed by pear (3.30%) and cherry (0.56%) and citrus (1.51%), respectively. This is due to the suitable climate and awareness among the growers. Apple is an important temperate fruit and a maximum area (47.91%) is available for its cultivation. The maximum area as well as production in Ladakh is under apple production followed by apricot in Leh and equally in Kargil. Ladakh contributes to an area of 1.10% to horticulture cultivation with 0.42% production (Rehman et al., 2020). In the dry fruit category, walnut is produced in highest quantity in the Kashmir region (63.41%), followed by Jammu region (32.79%). In Kashmir valley, the maximum area and production of fresh fruits are being produced from district Baramulla followed by Shopian, Kulgam, Kupwara, Anantnag, Pulwama, Budgam, Ganderbal, Bandipora, and Srinagar. In the Jammu region, Kathua is the top district in land area and production of fresh fruits, followed by Jammu followed, Rajouri, Poonch, Doda, Udhampur, Samba, Reasi, Ramban, and Kishtwar. From the Ladakh region, maximum production was recorded from Kargil followed by Leh.

In 2016–2017, Jammu & Kashmir contributed 2.11% to the horticulture production of India. The area expansion of about 1851 ha in the year 2016–2017 resulted in extra production of 287805MT as compared to the previous year. Most of the area available in Jammu & Kashmir is under apple production (162291MT), followed by pear (88329MT) and citrus (34192MT). In the Kashmir region, apricot production is at a third position under the fresh fruit category. Among dry fruits, walnut occupies maximum area and production followed by almond and peanut in both the UT's (Jammu & Kashmir and Ladakh).

In 2017–18, J&K contributes about 2.49% to the total horticulture production in India which is significantly (0.38%) higher than 2016–2017. The total fruit production of 2429822MT during 2017–18 comprising of 2141182MT of fresh fruits and 288640MT of dry fruits (Directorate of Horticulture, Kashmir). The area of 79938 ha under major horticulture crops in Jammu during, 2016–2017 and 118557 ha in 2017–2018, respectively are responsible for the expansion of 38619 ha. From this expanded area, 74173MT fruits were produced more in the Jammu region. Maximum area expansion of 54217 ha was brought under horticulture cultivation and the 391481MT fruits were produced from the newly cultivable area from the Kashmir region of UT of J& K. Most of the area available under horticulture sector in 2017–18 is in district Baramulla followed by Shopian, Kupwara, Kulgam, Anantnag, Budgam, Pulwama, Ganderbal, Bandipora, Jammu, Kathua, Rajouri, Srinagar, Udhampur, Doda, Samba, Poonch, Ramban, Kargil, Reasi, Leh, and Kishtwar. District Kishtwar of the Jammu region has a small area under fruit cultivation during 2017–18. District Baramulla is one of the leading districts in apple production (Sangral, 2015). Among fresh fruits, apple forms 87. 93% production followed by pear (4.18%) and mango (1.42%). In the case of dry fruits, walnut makes up 95.43% of production followed by almond (4.54%) and peanut (0.02%), respectively.

During 2018–19, the area of 331538 ha and the production of 2415421MT under horticulture in Jammu & Kashmir contributed about 2.20% to the overall production to India. The total area of 72.58 % and 27.42% occupied under fresh and dry fruits were brought under horticulture production and the maximum production of fresh (91.28%) and dry fruits (70.81%), respectively, produced from Kashmir valley of UT of Jammu & Kashmir during the year 2018–19. In the Jammu region, the percentage of (7.96%) under fresh and (29.13%) of dry fruits, produced from the Jammu region from the UT of J&K. Besides, the UT of Ladakh contributes only 0.76% fresh and 0.06% dry fruits to the newly carved UT of J&K.

**Figure 7:**
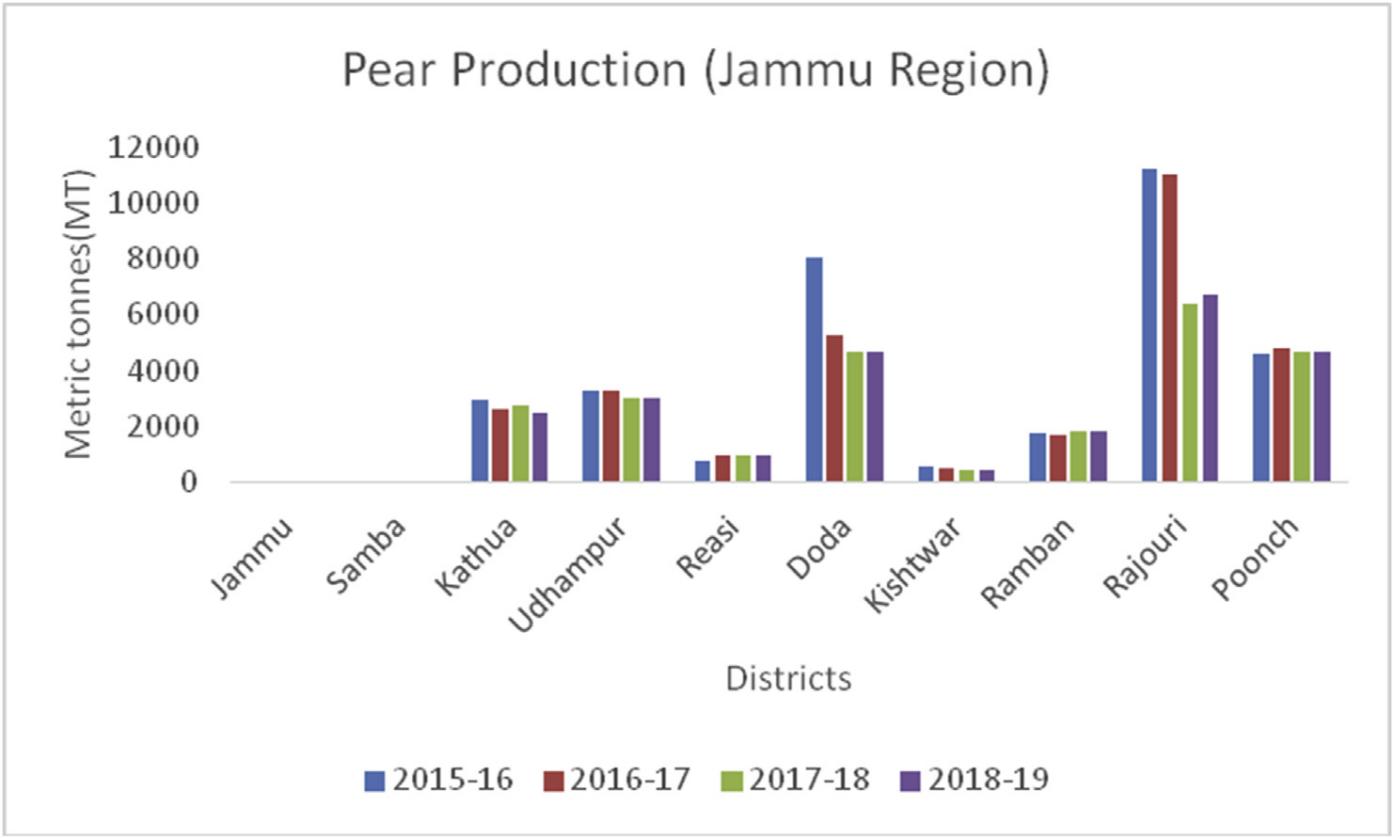
Pear production in metric tons (MT) from 2015-2019 in Jammu region of Union Territory of Jammu & Kashmir.

**Figure 8:**
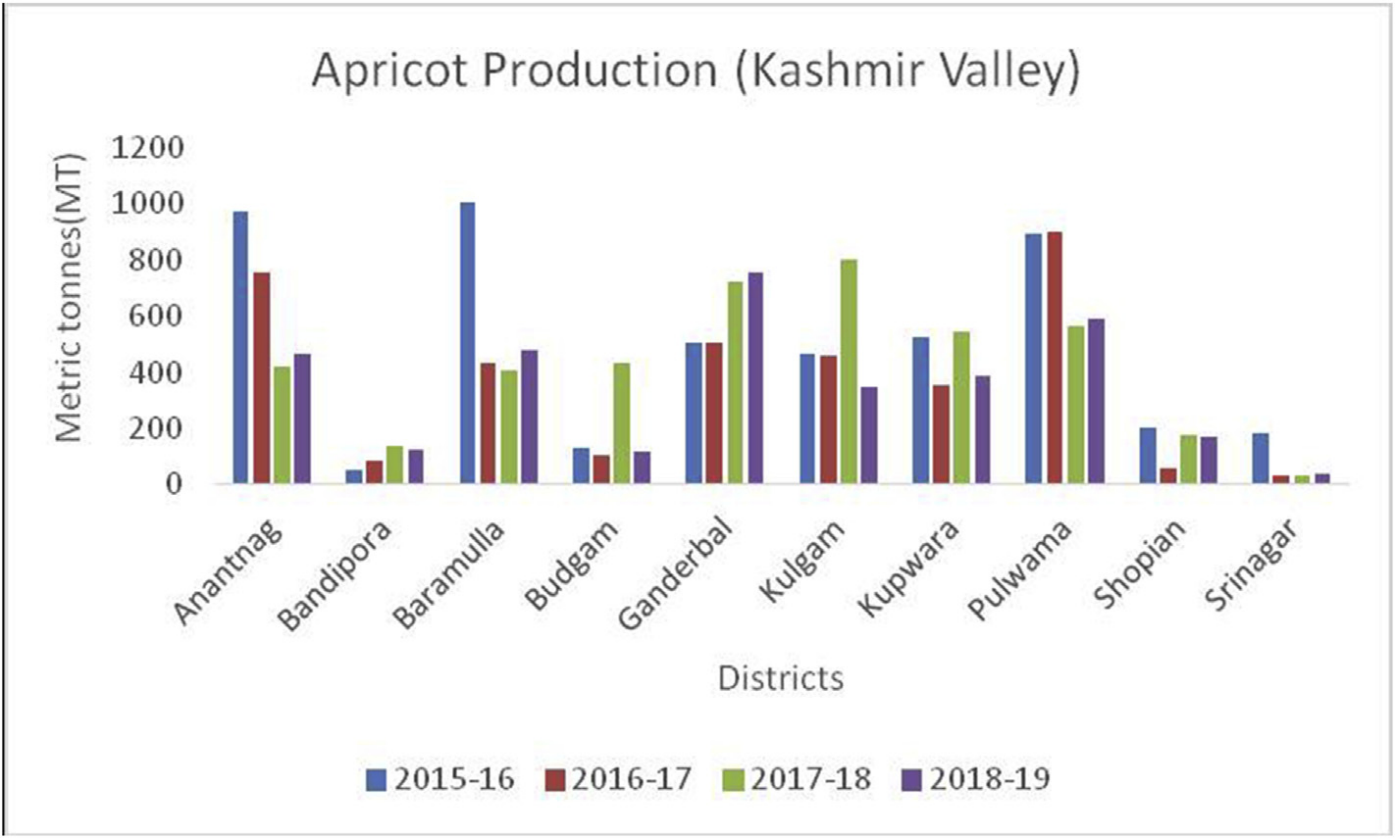
Apricot production in metric tons (MT) from 2015-2019 in Kashmir Valley of Union Territory of Jammu & Kashmir.

**Figure 9:**
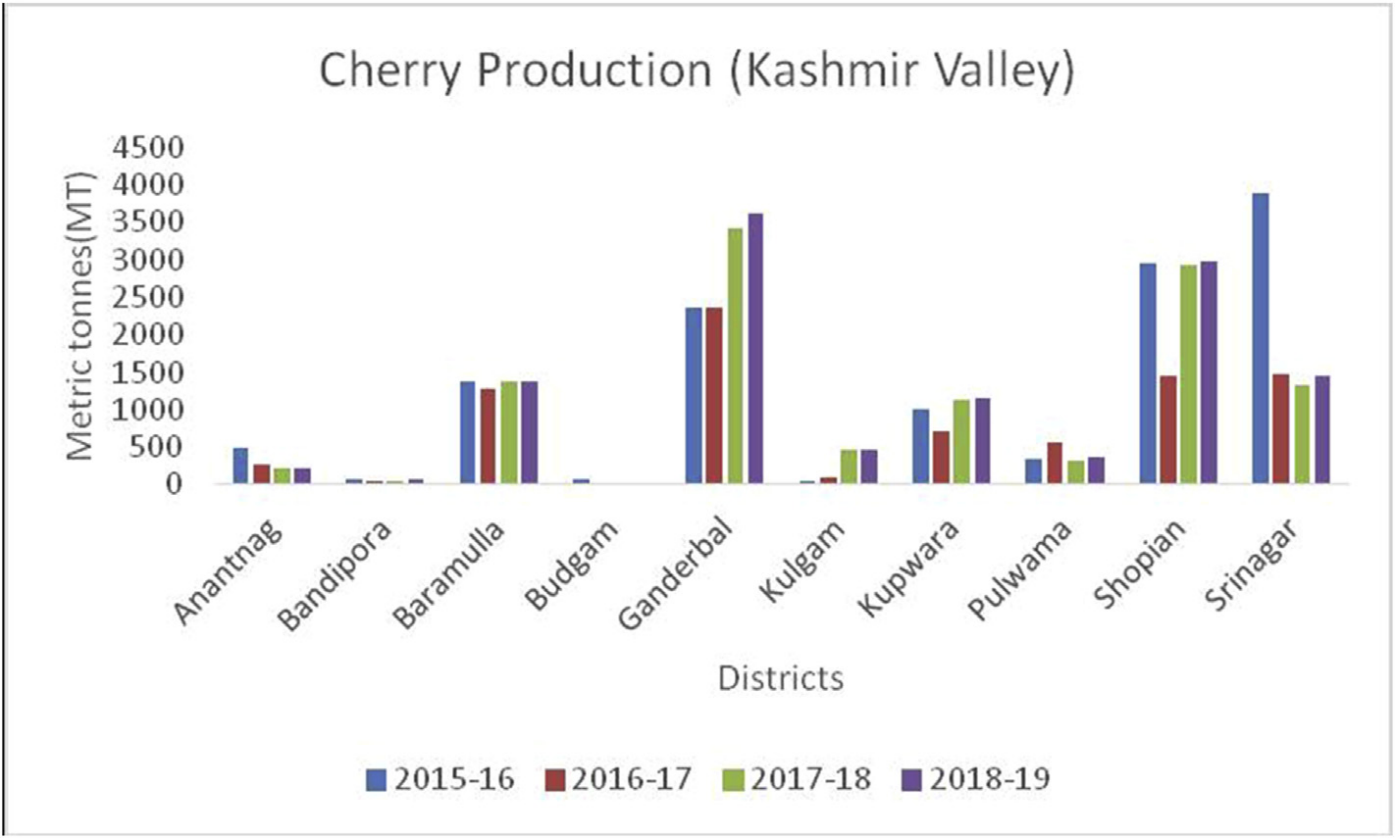
Cherry production in metric tons (MT) from 2015-2019 in Kashmir Valley of Union Territory of Jammu & Kashmir.

Apple, (*Malus spp*) a member of the Rosacea family is an edible temperate fruit, originated from Central Asia and is cultivated all over the world. China ranked first in apple production (41000000MT) during 2019–20, followed by European Union (11477000MT), USA (4821000MT), Turkey (3000000MT) and India (2370000MT) (Sahbandeh, 2020). Red delicious, Golden delicious, Mcintosh, Jonagold, Cameo, Empire, Gala, Granny smith, Braeburn, and Fuji varieties are cultivated in Jammu & Kashmir, Himachal Pradesh, Uttar Pradesh, Uttaranchal, Arunachal Pradesh, and Nagaland. Harvesting season is strongly influenced by climate and is normally practiced in September to early October. During 2015–16 to 2018–19, district Baramulla is leading in apple production followed by Kupwara, Shopian, and Kulgam in the Kashmir region (Figure 7). Doda is the leading district from Jammu in apple production followed by Ramban and Kishtwar (Figure 8). Leh district recorded maximum apple production during, 2018–19 (Figure 9). Apple fruits are rich in sugars, minerals, dietary fiber, ascorbic acid, and phenolics (Bondonno et al., 2017), and have resistance to various diseases like Parkinson's disease, Alzheimer's disease, cataract, gallstone, and cancer (USDA, 2020). Among 22 cultivars of apple as studied by Kumar et al. (2018), the maximum Vitamin C content was recorded in red cultivars, "Starkrimson" and "Oregon Spur II" and the highest content of sucrose recorded in non-red cultivar "Granny Smith" and lowest in red cultivar "Royal Delicious".

**Figure 10:**
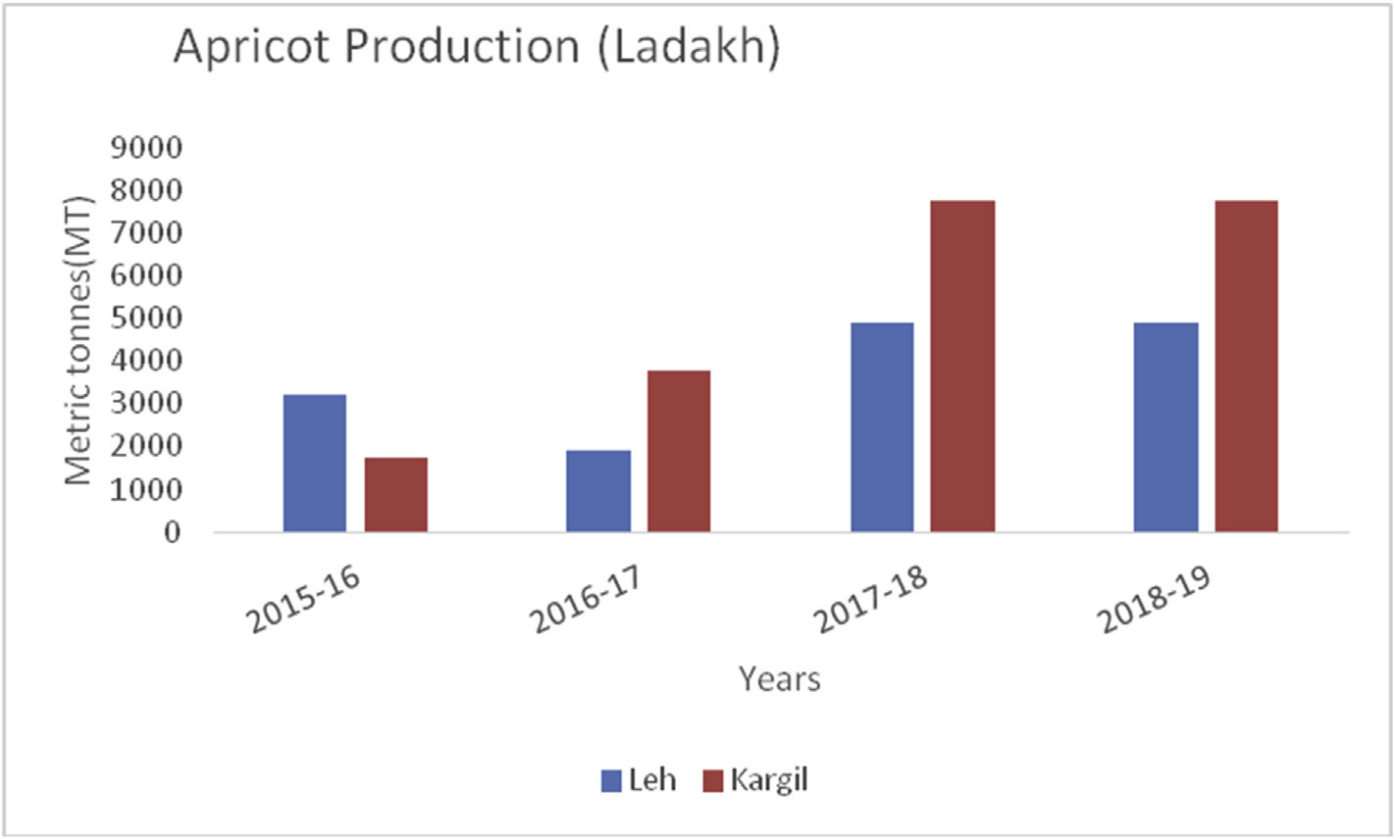
Apricot production in metric tons (MT) from 2015–19 from Union Territory of Ladakh.

**Figure 11:**
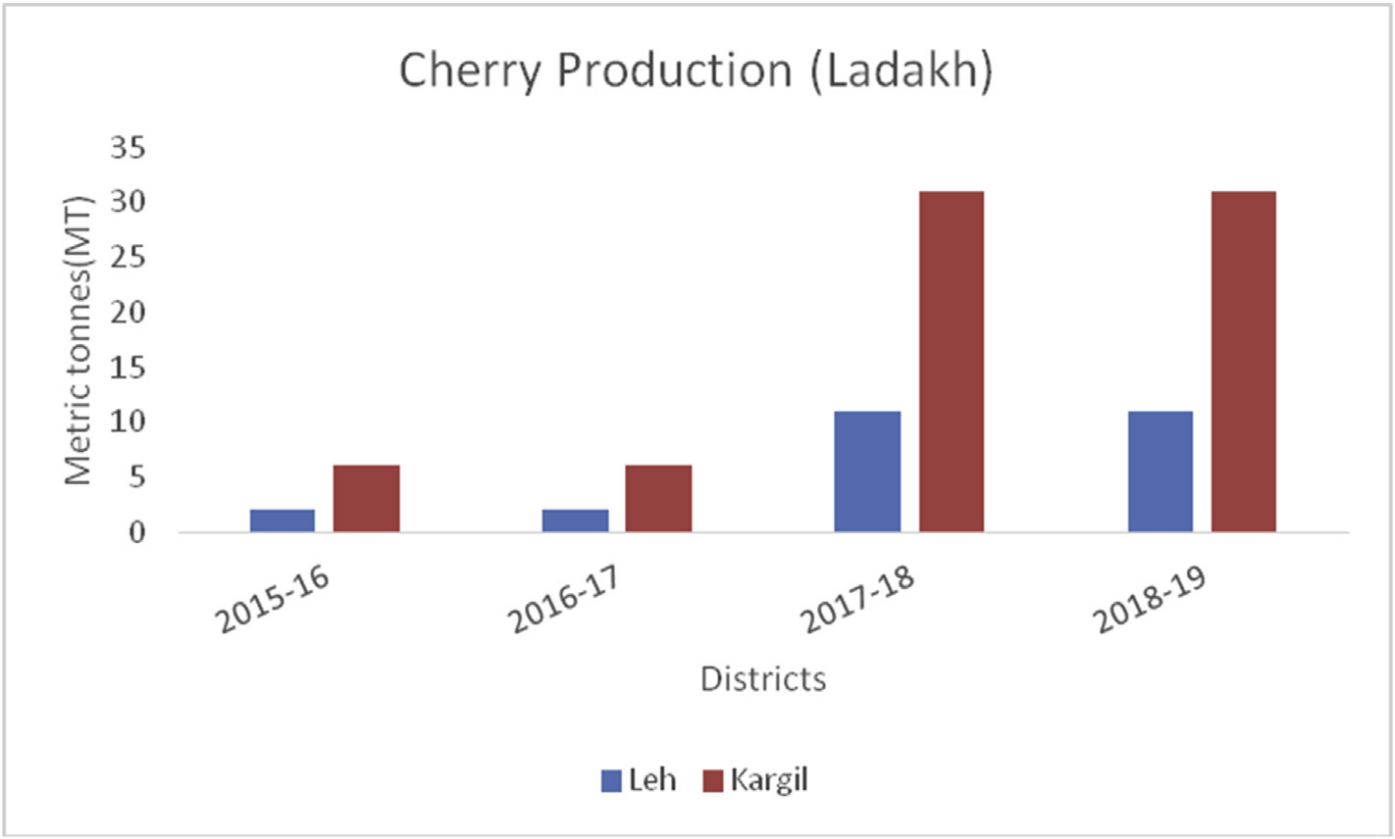
Cherry production in metric tons (MT) from 2015–19 from Union Territory of Ladakh.

Pear (*Pyrus spp*) belongs to the Family: Rosaceae is an important crop of a temperate region with delicious fruit with a pleasant flavor native to the Northern Hemisphere. Pears are a source of many nutrients, including fiber, vitamin C, potassium, calcium, phosphorus, iron, antioxidants, fructose, rich in proteins, and sorbitol (Reiland and Slavin, 2015). China occupy the leading position in pear production (17000MT) followed by European Union (2184MT), USA (658MT), Argentina (550MT), Turkey (490MT), South Africa (407MT), India (340MT) and Japan (275MT) during 2019–20 (USDA, 2020). In India, it is cultivated in Punjab, Himachal Pradesh, Jammu & Kashmir, Uttar Pradesh, and Tamil Nadu and some varieties are cultivated in subtropical regions. About 3000 varieties are grown worldwide; some important varieties are William, Kashmir nakh, Vicar of wakefield, Beuree D, Amanalis, Goshbagu, and Beurre hardy. Some important species of pear are *P. pyrifolia*, *P. communis*, *P. serotina*, and *P. pashia*. During the crop year 2018–19, maximum production of pear were reported from district Budgam (17685MT) followed by Baramulla (9971MT), Pulwama (8691MT), Kupwara (5832MT), Kulgam (5771MT) Ganderbal (5232MT), Shopian (2504MT), Anantnag (2126MT), Srinagar (2060MT) and Bandipora (1281MT), respectively (Figure 10). While as, Rajouri, Poonch, and Doda are cultivating apples from Jammu (Figure 11).

**Figure 12:**
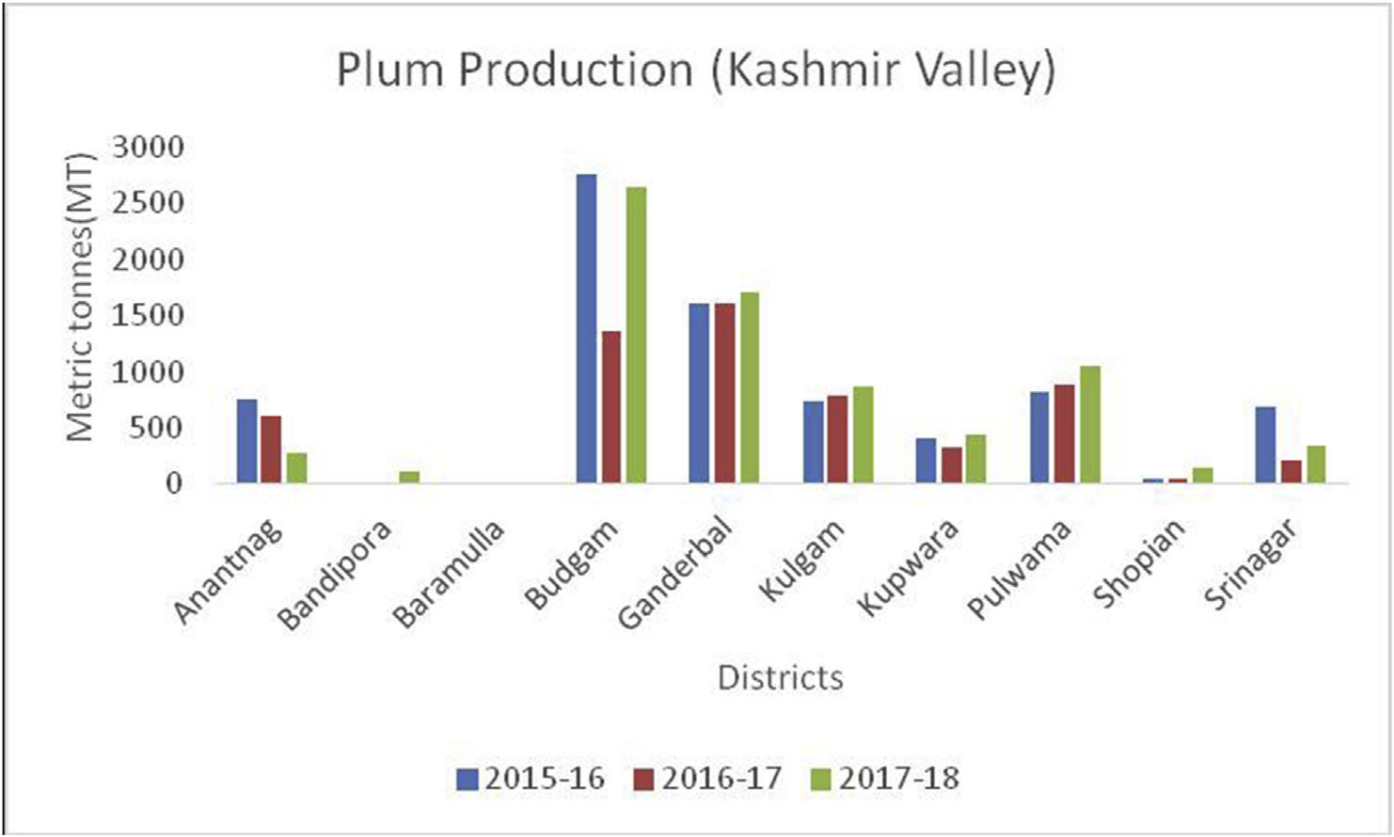
Plum production in metric tons (MT) from 2015-2019 in Kashmir Valley of Union Territory of Jammu & Kashmir.

**Figure 13:**
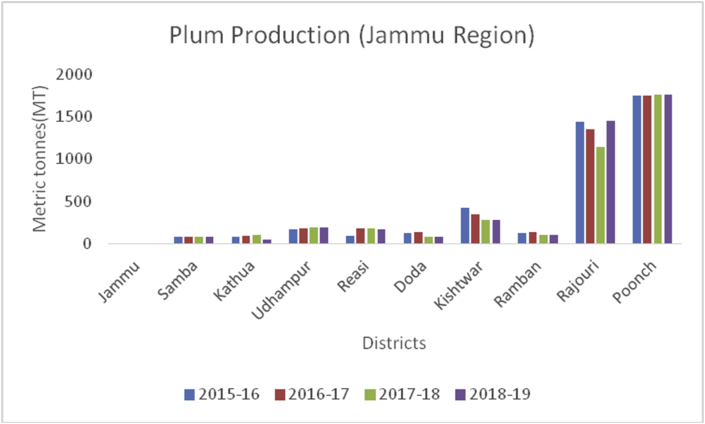
Plum production in metric tons (MT) from 2015-2019 in Jammu region of Union Territory of Jammu & Kashmir.

**Figure 14:**
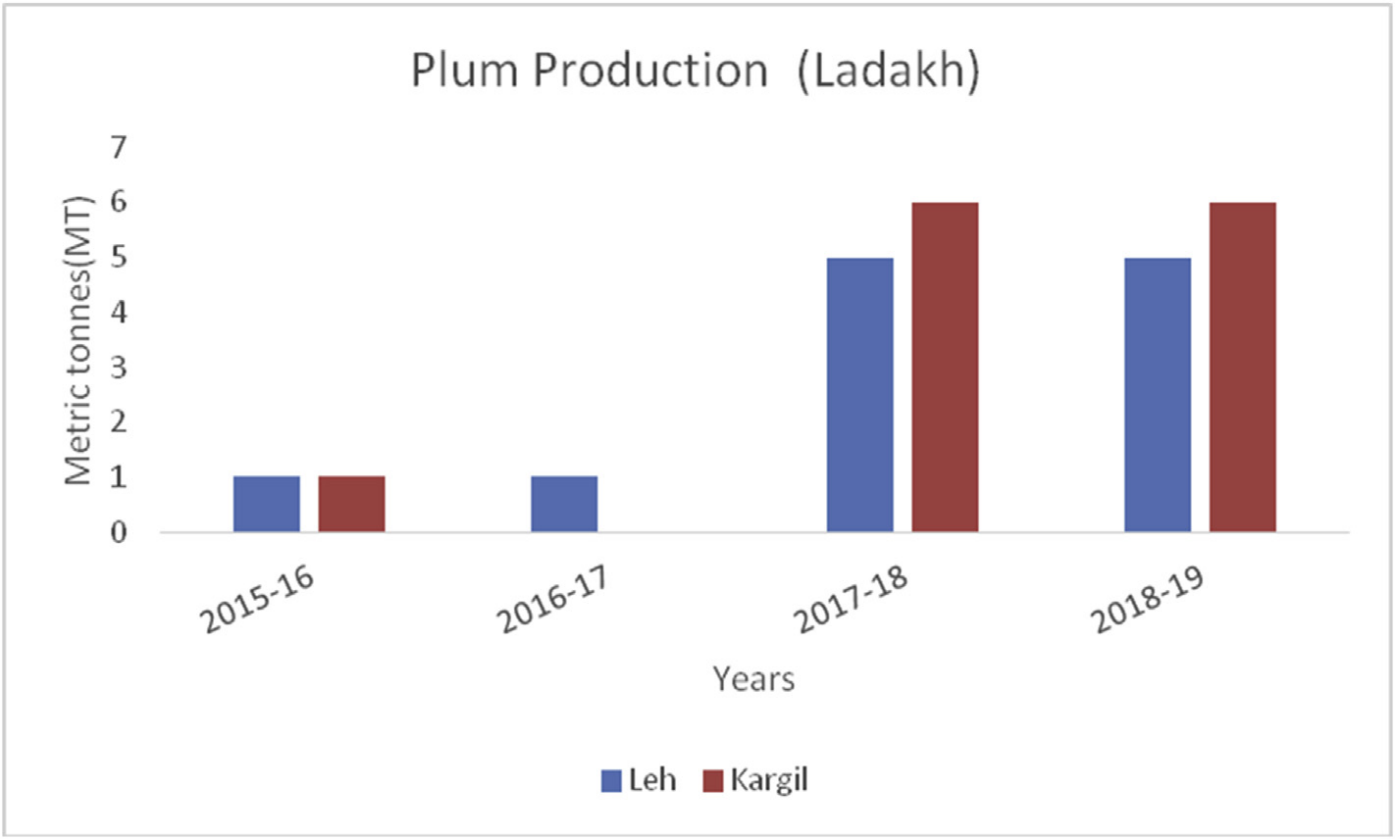
Plum production in metric tons (MT) from 2015–19 from Union Territory of Ladakh.

Apricot, (*Prunus armeniaca*): (Family: Rosaceae) is a juicy, round stone fruit with yellow-orange flesh. Apricot trees grow well in higher altitudes and long shelf life. Apricot first originated from China and now cosmopolitan in distribution. Apricots are rich in vitamins (A, C, K, B6), sugar (more than 60%), proteins (8%), crude fiber (11.50%), crude fat (2%), total minerals (4%), sodium, potassium, calcium, and iron (Fatima et al., 2018). Hence are good against anemia and useful for eyesight and strengthening the human body. At global level, Turkey is the main producer of Apricots (795768MT), accounting to 18% of world apricot production followed by Iran (460000MT), Uzbekistan (365000MT), Algeria (269308MT), Italy (247146MT) and Pakistan (192500MT) (FAOStat.org). The annual production of apricots from 2015–16 to 2018–19 are presented (Figures 12 and 13) from Jammu &Kashmir. Ladakh is the home of the world's sweetest apricots (Sing, 2013; Angmo et al. 2017) with an annual production of 12686MT during the crop year 2018–19 (Figure 14).

**Figure 15:**
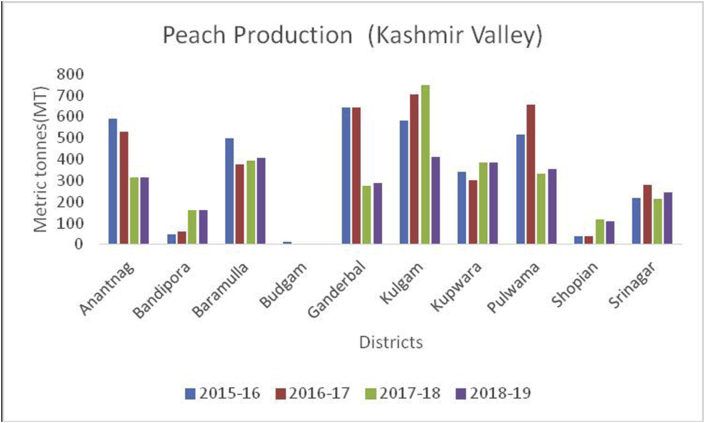
Peach production in metric tons (MT) from 2015-2019 in Kashmir Valley of Union Territory of Jammu & Kashmir.

Cherry (*Prunus avium*): (Family: Rosaceae), are small stone fruits rich in, potassium, Vitamin A and C, sodium, carbohydrate, carbohydrates, magnesium, calcium, iron, protein, fat and are rich in antioxidants and anti-inflammatory compounds (Vicente et al., 2009); Kelly et al., 2018). Globally, 2317958 tons of cherries are produced yearly. Turkey is the largest cherry producer in the world with an annual production of 599650 tons, followed by the United States of America with 288480 tons. Yearly, India produced (10936MT) of cherry annually and is ranked at 26 (www.atlasbig.com). Nearly 2,713 ha of land are under cherry cultivation in both the union territories. Jammu & Kashmir and Ladakh have been contributing a significant role in cherry production. The area of 2713 ha in Jammu &Kashmir and Ladakh is under cherry with a production of 11789MT.Besides, cherry is grown in some parts of Srinagar, Baramulla, and Shopian of south Kashmir (Figures 13 and 15), and in district Ganderbal of north Kashmir. Cherry harvesting season begins from mid-May and lasts up to the 1^st^ week of July (Hindustan Times, 2019).

**Figure 16:**
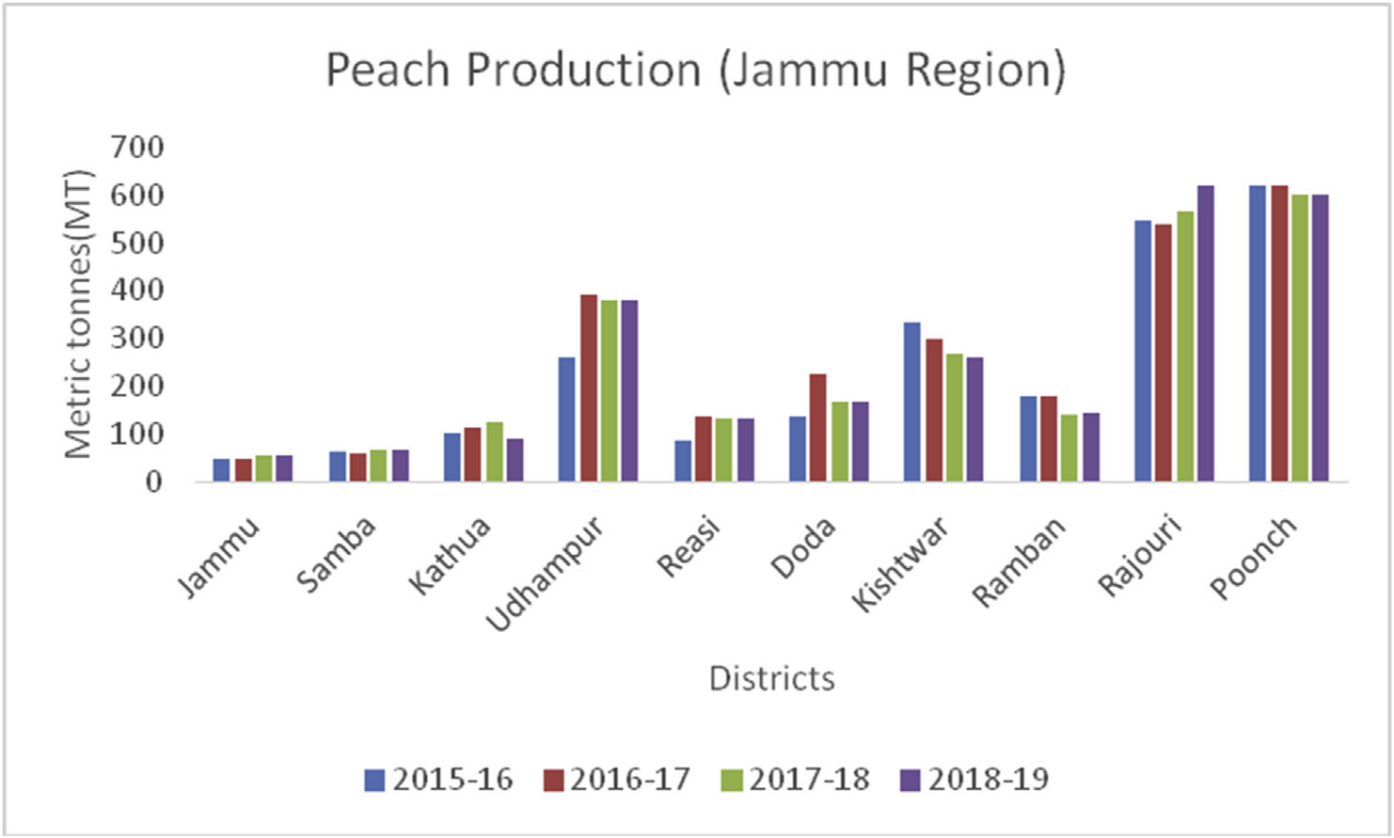
Peach production in metric tons (MT) from 2015-2019 in Jammu region of Union Territory of Jammu & Kashmir.

**Figure 17:**
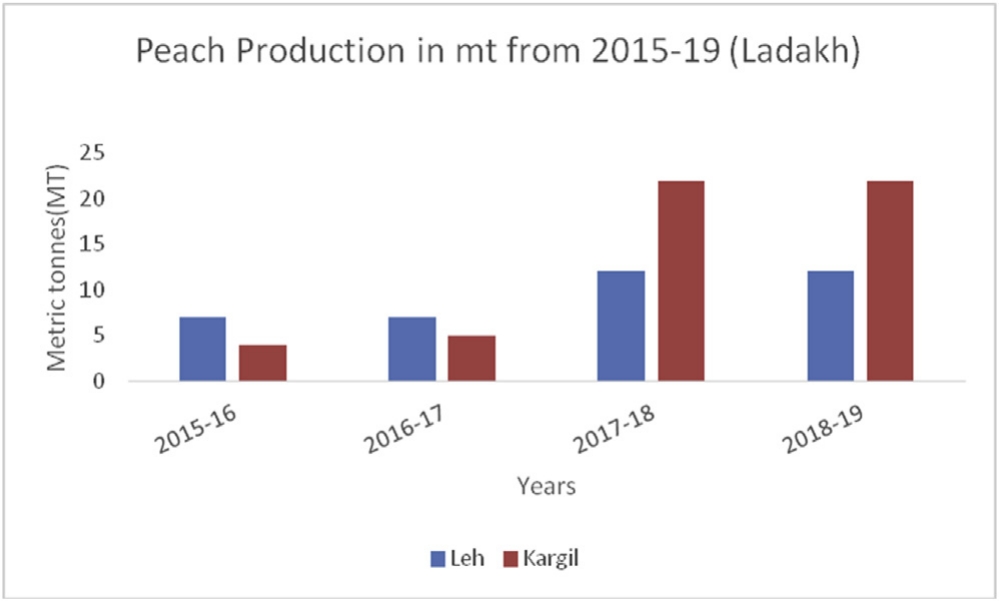
Peach production in metric tons (MT) from 2015–19 from Union Territory of Ladakh.

**Figure 18:**
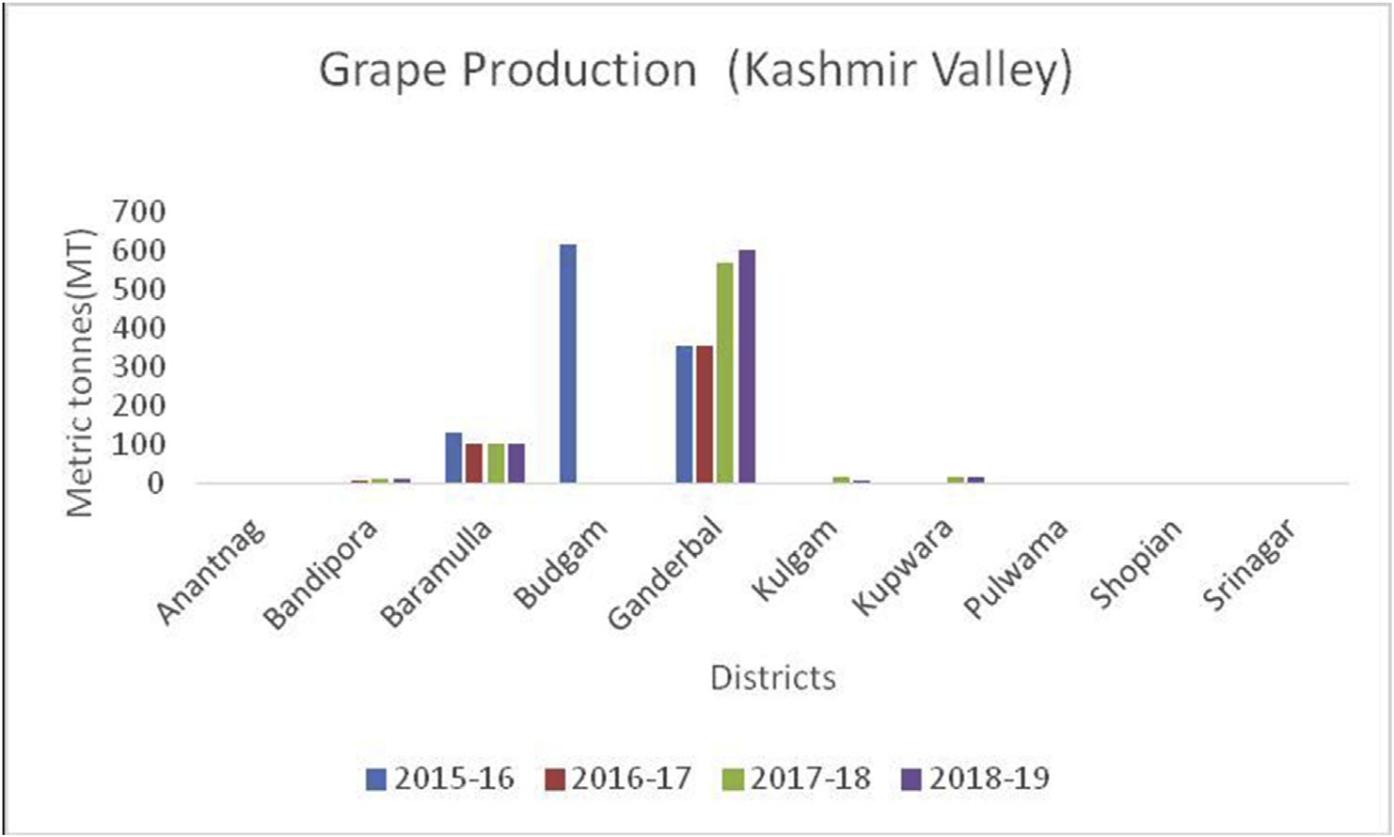
Grape production in metric tons (MT) from 2015-2019 in Kashmir Valley of Union Territory of Jammu & Kashmir.

Plum (*Prunus domestica*):(Family: Rosaceae), is paraben-free, sweet stone fruit, rich in proteins, potassium, and vitamin C. They are rich in antioxidants and helpful in treating cancer, diabetes, obesity, and blood pressure (Vicente et al., 2009). China is the leading producer for plum production (6663165MT) in the world followed by Serbia (391485MT), Romania (424068MT), Chile (300000MT), Turkey (297026MT), Iran (295000MT), USA (229731MT) and India with (215000MT) respectively, during 2014–15 (www.mapsofworld.com) (FAO Stat database, 2014). District Budgam is one of the leading producers of Plum followed by Ganderbal and Pulwama in the Kashmir region. Poonch and Rajouri from Jammu region Figures 16, 17 and 18) are important districts for plum production from 2015–16 to 2018-19.

**Figure 19:**
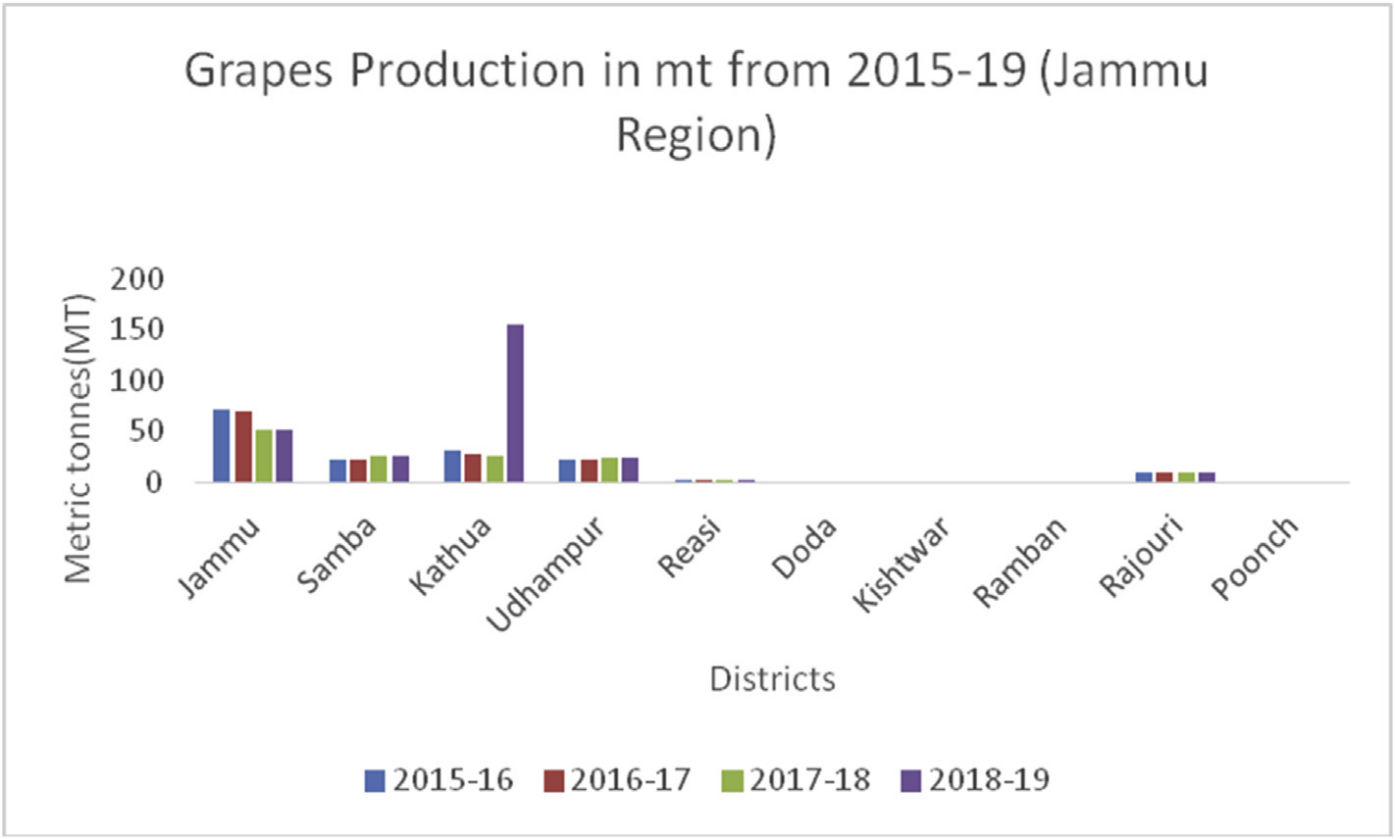
Grape production in metric tons (MT) from 2015-2019 in Jammu region of Union Territory of Jammu & Kashmir.

**Figure 20:**
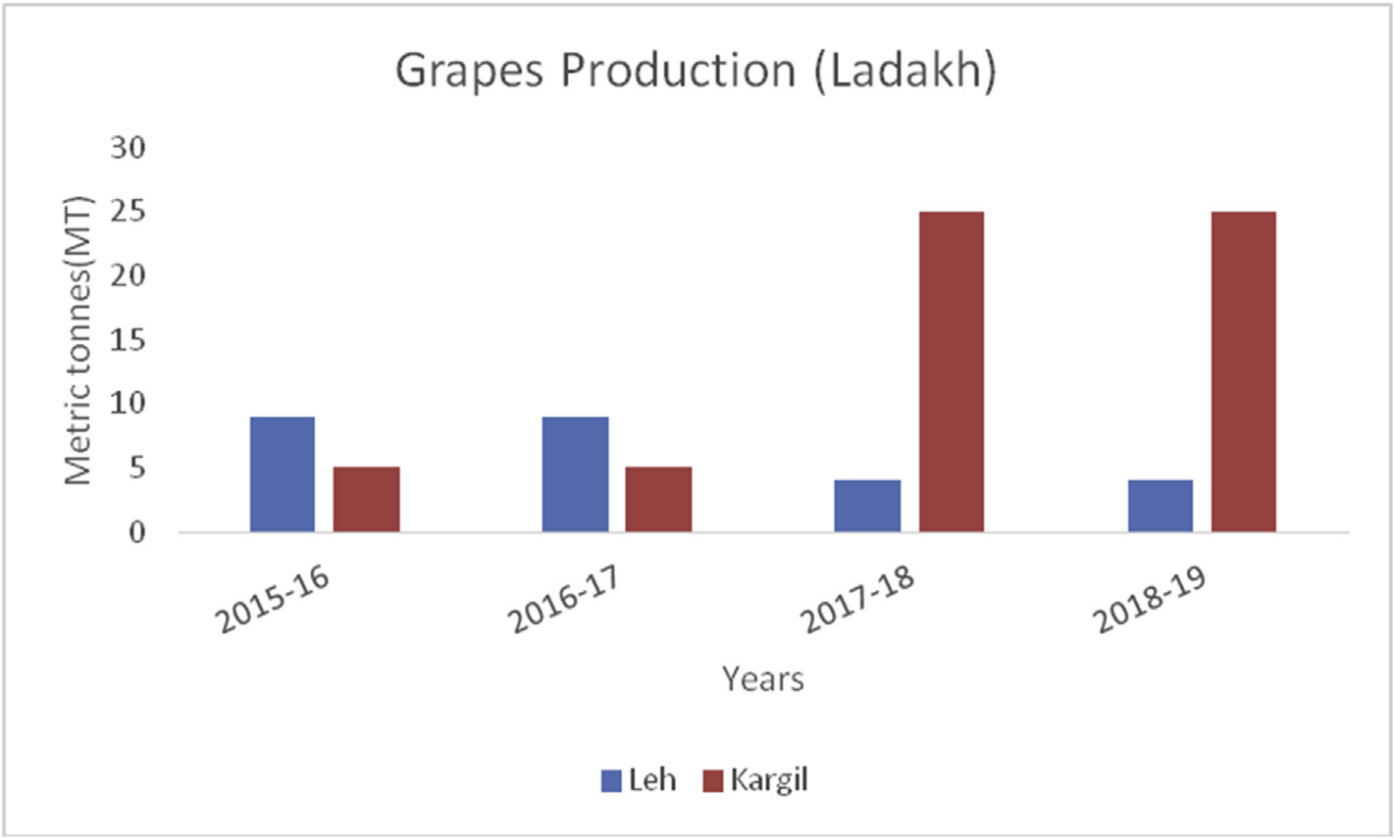
Grape production in metric tons (MT) from 2015–19 from Union Territory of Ladakh.

**Figure 21:**
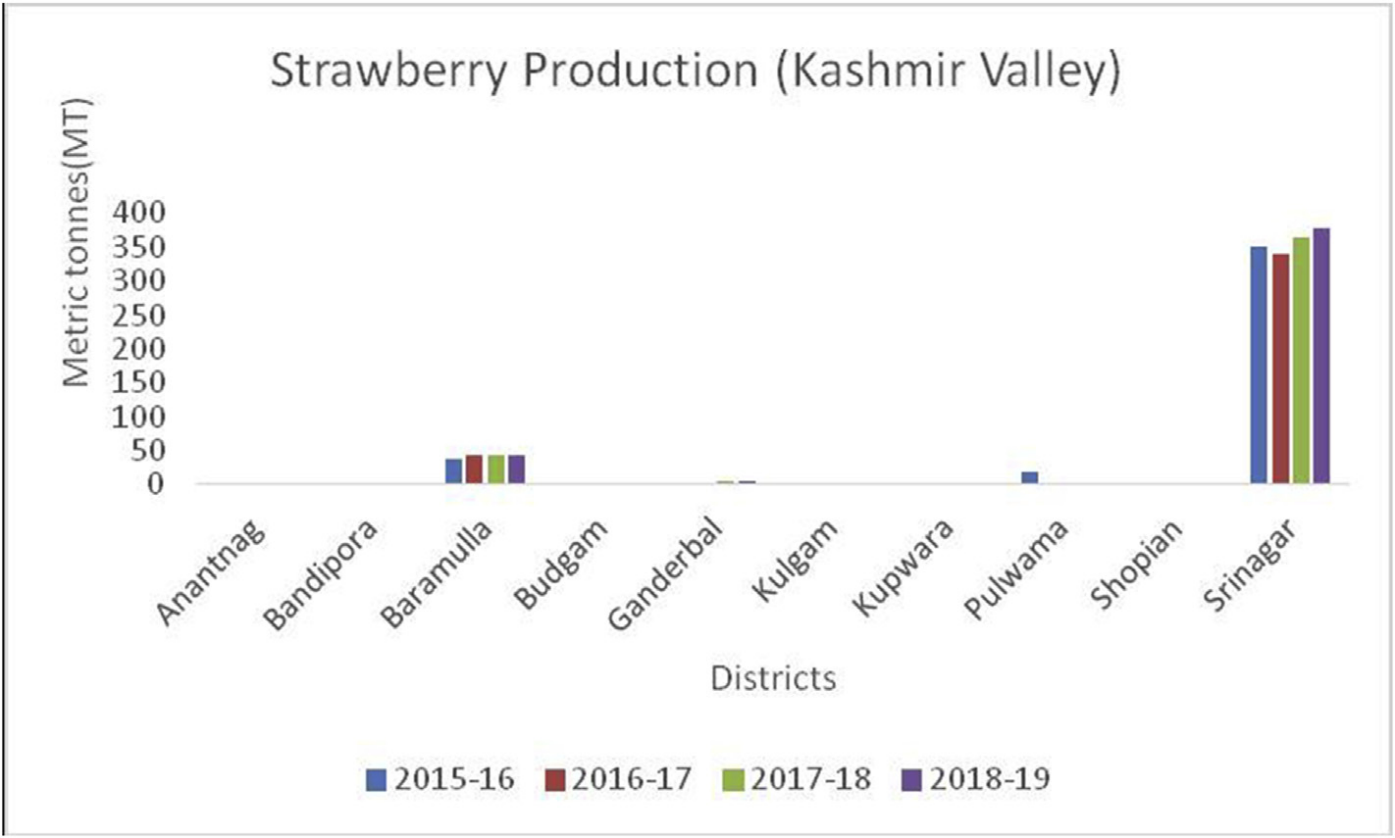
Strawberry production in metric tons (MT) from 2015-2019 in Kashmir Valley of Union Territory of Jammu & Kashmir.

Peach (*Prunus persica*): (Family Rosaceae), is a soft, juicy, and fleshy stone fruit, native to Northwest China. The inner flesh of a peach is white to yellow or simply orange. Peaches are rich in vitamin A and C and minerals/100g like potassium (190g), calcium (6g), phosphorous (20g), manganese (0.061g), and magnesium (9g). They are also rich in carotenoids and contains 1.5% dietary fiber (Vicente et al., 2009). Eating peaches may help a person in the reduction of cancer risk, preserving skin health, and to boost the hemoglobin level. China is the leading producer with a maximum production of (15000MT) followed by the European Union (4118MT), Turkey (830MT), the USA (717MT), and Argentina (226MT) during 2019–20 (USDA, 2020). In India, peaches are mainly grown in the North-Western States of J&K, H.P, and U.P hills. The main districts categorized on area and production under peach cultivation are Kulgam followed by Ganderbal and Pulwama (Figures 19, 20 and 21) from 2015-2019.

**Figure 22:**
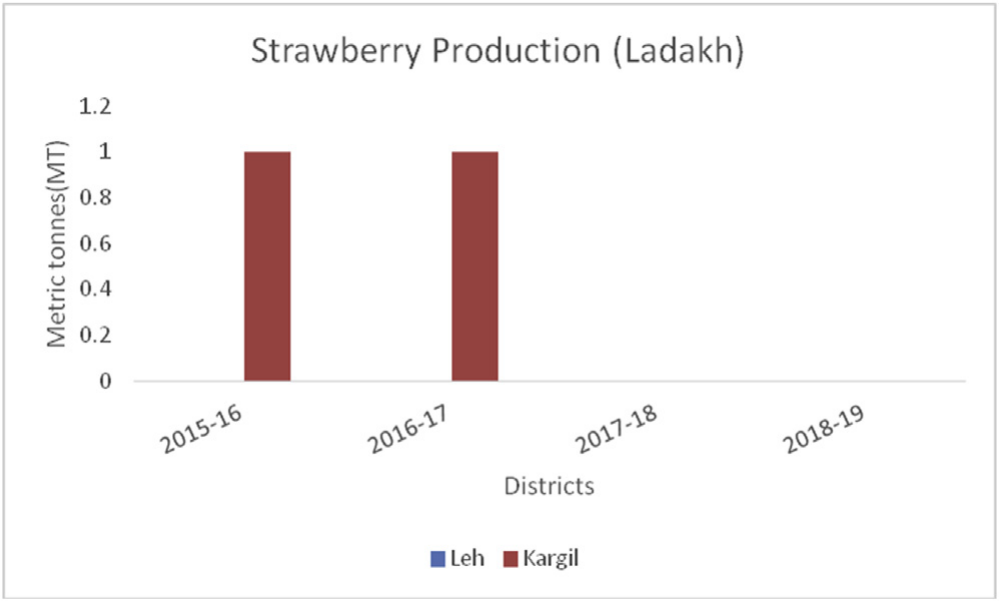
Strawberry production in metric tons (MT) from 2015–19 from Union Territory of Ladakh.

**Figure 23:**
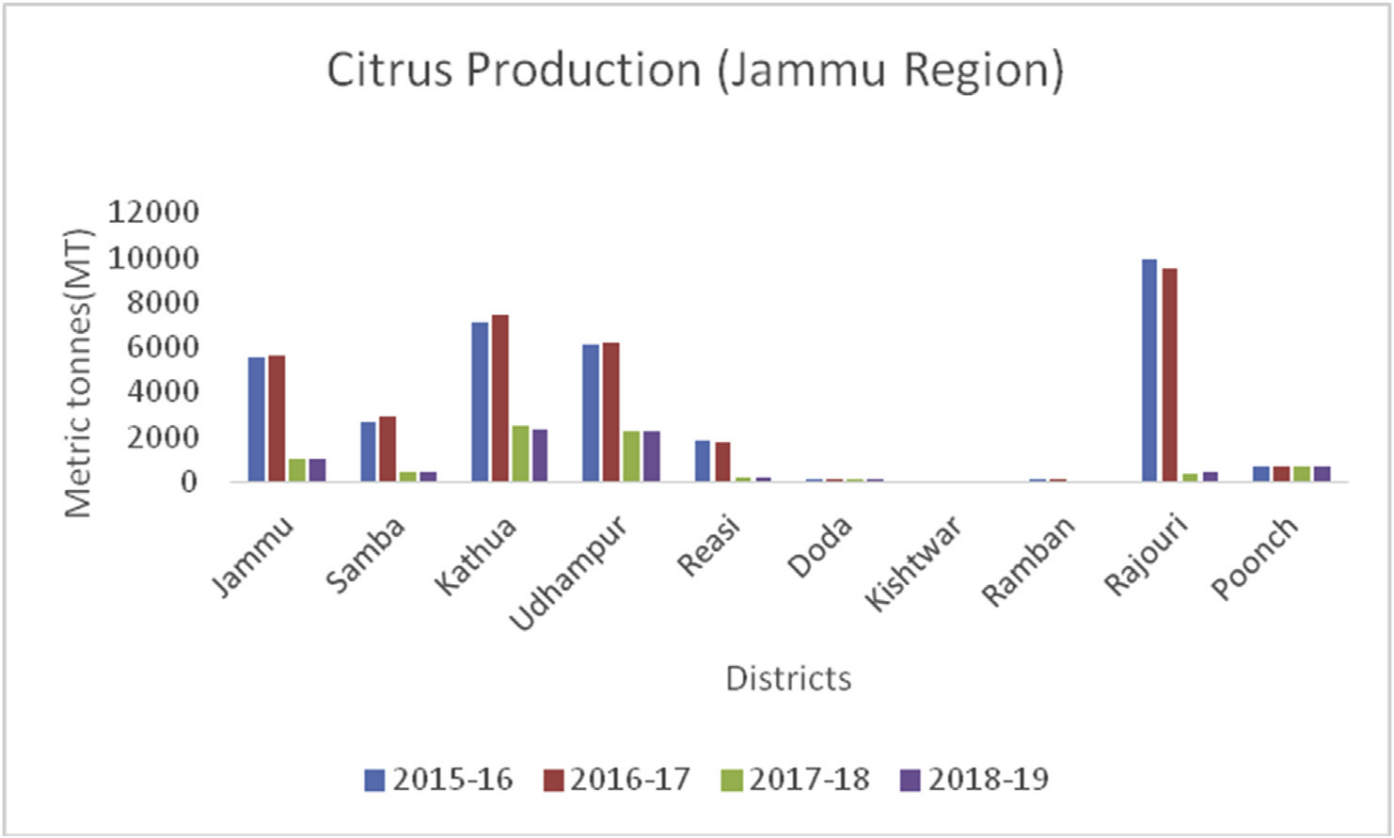
Citrus production in metric tons (MT) from 2015-2019 in Jammu region of Union Territory of Jammu & Kashmir.

**Figure 24:**
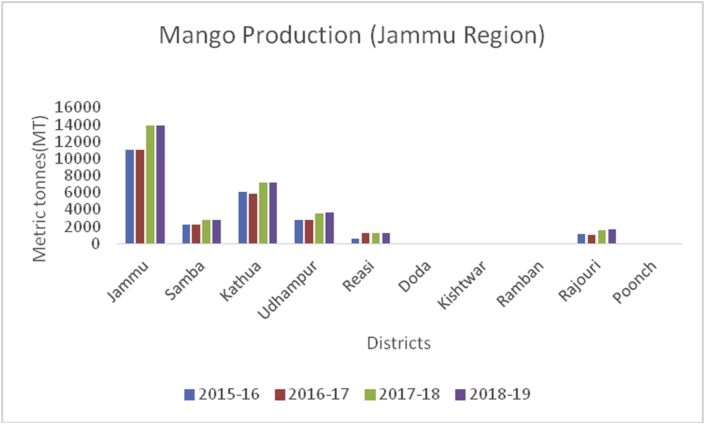
Mango production in metric tons (MT) from 2015-2019 in Jammu region of Union Territory of Jammu & Kashmir.

Grapes (*Vitis vinifera*) (Family: Vitaceae), are deciduous woody vines and the fruits are rich in Vitamin A, B6, C, and K, carbohydrates, potassium (191mg/100g), sodium (2mg/100g), magnesium (7mg/100g), calcium (10mg/100g) and phosphorous (20mg/100g) (Vicente et al., 2009). Grapes are useful in preventing chronic diseases, decrease blood sugar levels, and protect against diabetes due to their micro-bacterial, antioxidant, and anti-inflammatory perspectives (Imran et al., 2017). Globally, China is the leading producer in grape production from 2014–15 to 2019–20 with an average production (10800MT) followed by India (3000MT), Turkey (1950MT), Uzbekistan (1626MT), and European Union (1376MT) during the year 2019–2020 (USDA, 2020). Among the states in Indian Union, Maharashtra is the leading producer of grapes followed by Karnataka and Tamil Nadu. Among the states of India, the Nasik district of Maharashtra is the biggest supplier and famous for grapes, locally called the wine capital of India. Ganderbal district leading in the grape production (606MT) followed by Baramulla and Kupwara of Kashmir valley (Figure 22) during 2018–19. While, in the Jammu region, district Kathua is the leading district in grape production (156MT) followed by Jammu (51MT) in the UT of J&K (Figure 23). The UT of Ladakh recorded (29MT) of grape production and the maximum production of (25MT) recorded from Kargil district during 2018–19 (Figure 24).

**Figure 25:**
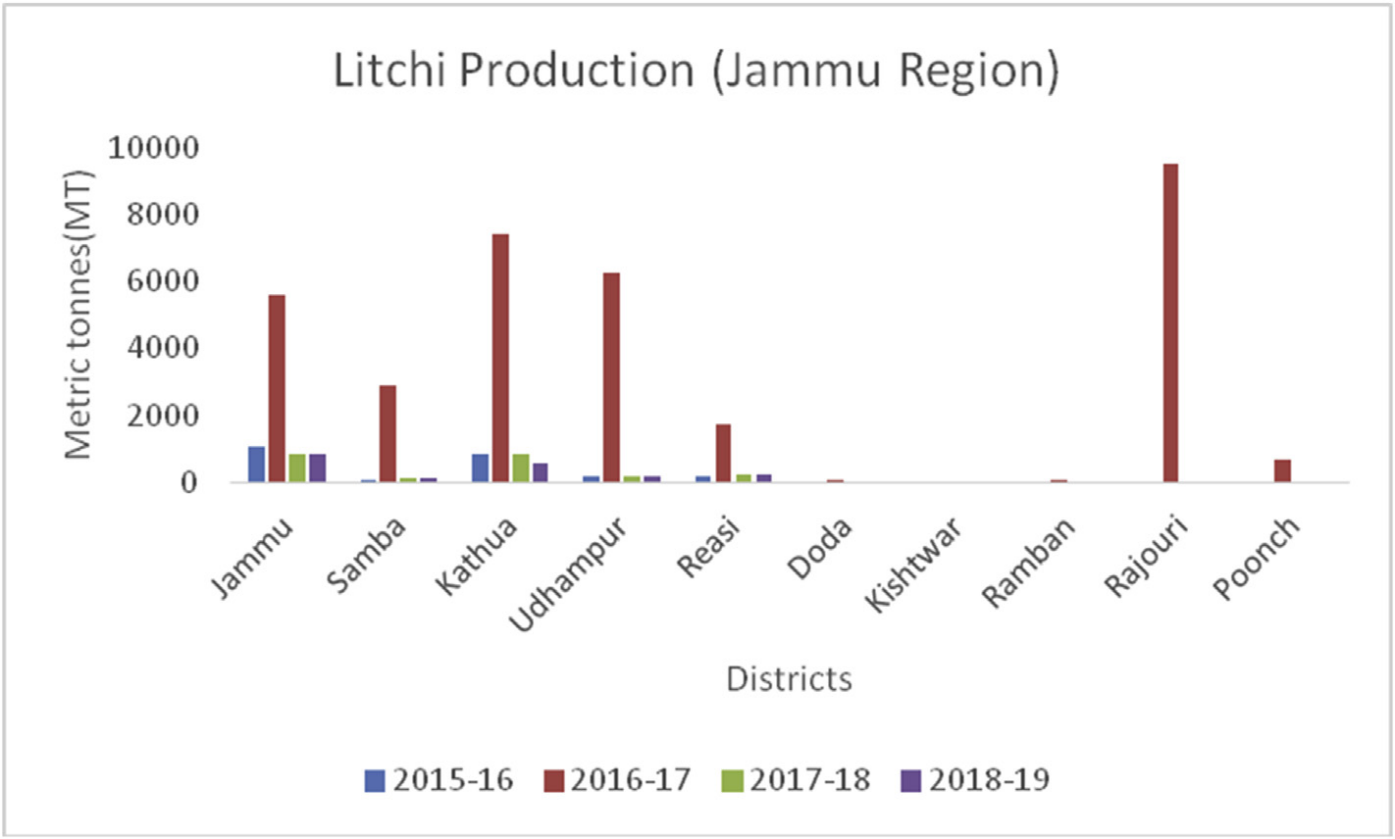
*Litchi* production in metric tons (MT) from 2015-2019 in Jammu region of Union Territory of Jammu & Kashmir.

**Figure 26:**
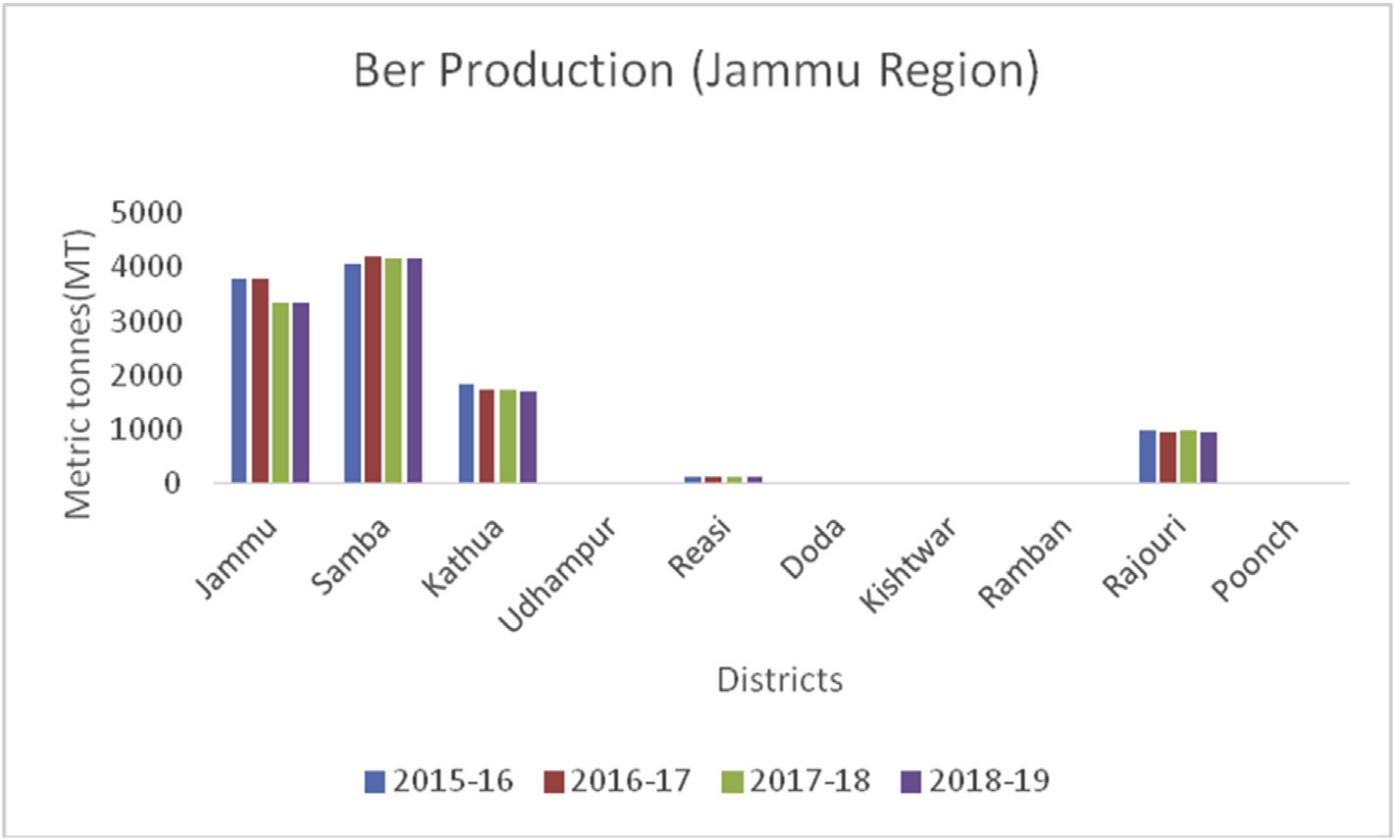
Ber production in metric tons (MT) from 2015-2019 in Jammu region of Union Territory of Jammu & Kashmir.

Strawberry (*Fragaria ananassa*): (Family Rosaceae), is heart-shaped fruit and widely appreciated for its characteristic aroma, bright red color, juicy texture, and sweetness. Strawberries are rich source of dietary fiber (2.0%), vitamin C (57mg/100g), potassium (153mg/100g), magnesium (13mg/100g), iron (0.41mg/100g), calcium (16mg/100g), sodium (1 mg/mg), and phosphorous (24mg/100g). USA is the global leader of strawberry production (1855196MT) during 2014–15, followed by Mexico (489198MT), Turkey (479354MT), Spain (393475MT) and (Egypt 328864MT) (www.mapsofworld.com). These fruits protect the heart, lowers blood pressure, and guard against cancer. Srinagar district is the leading producer in strawberry production (Figures 25 and 26).

**Figure 27:**
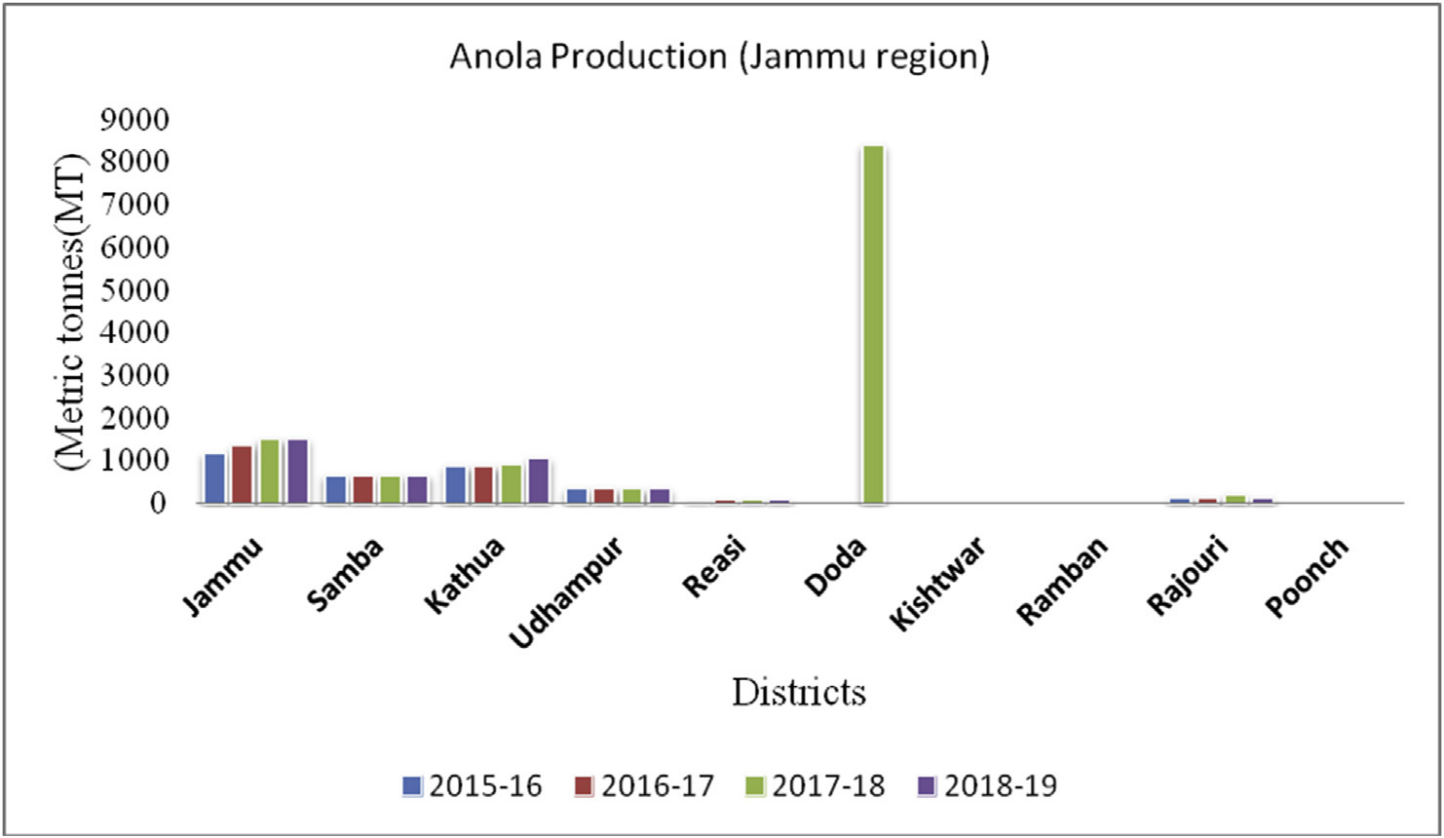
Aonla production in metric tons (MT) from 2015-2019 in Jammu region of Union Territory of Jammu & Kashmir.

*Citrus spp* is a juicy fruit with a sharp taste, and native to South Asia, East Asia, Southeast Asia, Melanesia, and Australia. Citrus belongs to the family Rutaceae. Brazil is the largest producer of citrus in the world followed by USA, China and Spain. Citrus is the most traded horticultural product in the world . They are a rich source of vitamin C and A, carotenoids, folate, fiber, flavonoids, and limonoids. It improves indices of antioxidant status, insulin sensitivity, and cardiovascular health (Turner and Burri, 2013). Brazil is the top exporter of orange juice in the world. Globally, China is the second leading country in citrus production (19.6MT) followed by the USA (10MT) and Mexico (6.8MT), respectively (Misachi, 2017). In Kashmir valley, citrus is cultivated in two districts (Kupwara and Baramulla) with an annual production of 28MT and 1MT during 2018–19, respectively. However, in the Jammu region, Kathua is the leading district (Figure 27) in citrus production (2308MT), followed by Udhampur (2266MT) and Jammu (970MT).

**Figure 28:**
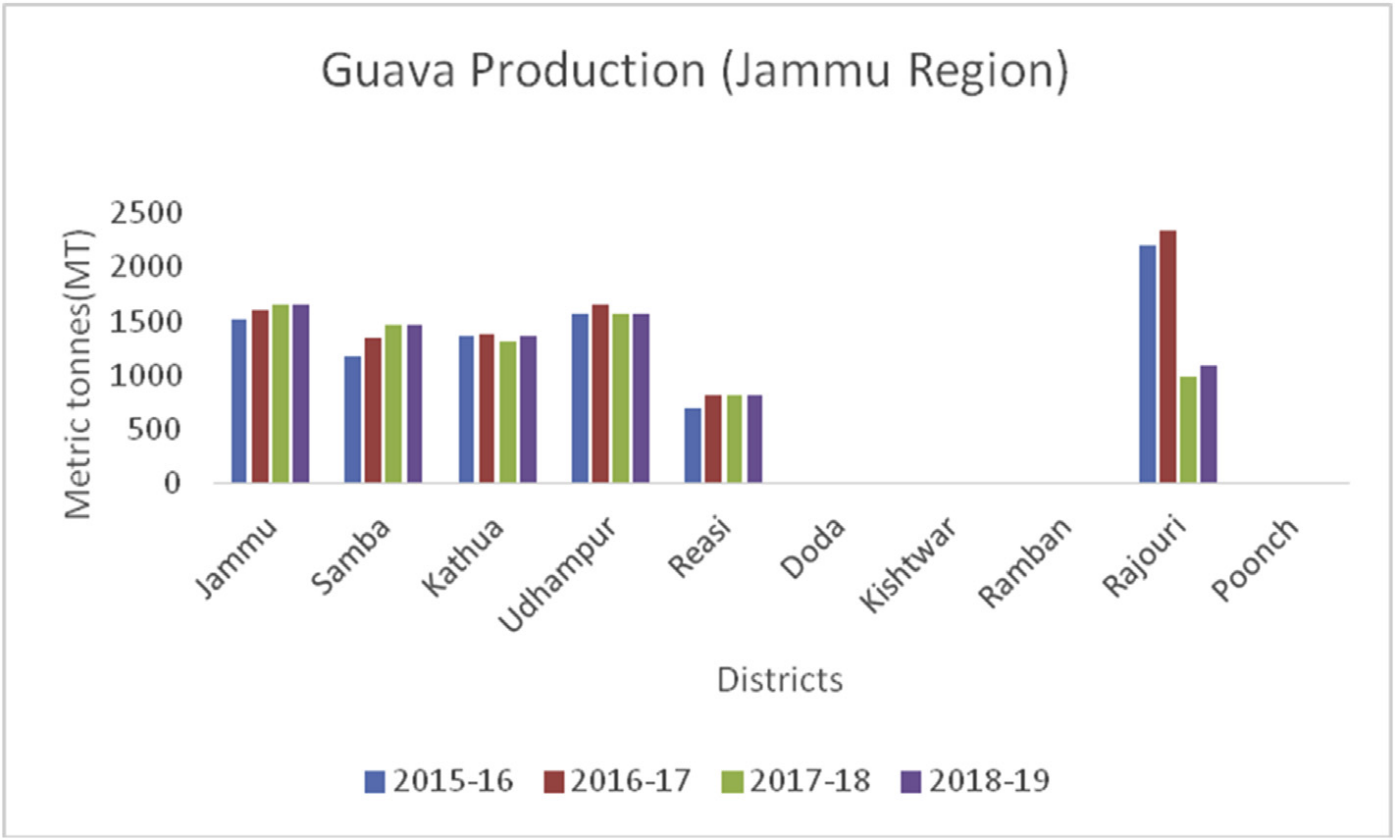
Guava production in metric tons (MT) from 2015-2019 in Jammu region of Union Territory of Jammu & Kashmir.

Mango, *Mangifera indica* known as the king of the fruits, is a juicy stone fruit (drupe) belonging to the Anacardiaceae family. Mangoes are native to South and Southeast Asia, and widely grown across the world. Mangoes are rich in minerals such as potassium (156mg/100g), calcium (10mg/100g), magnesium (9mg/100g), phosphorous (11mg/100g) and sodium (2mg/100g). They are rich in fiber, carotene, and carotenoids (Vicente et al., 2009). Mango improves digestion, promotes gut health, boosts immunity, lowers cholesterol, improves eyesight, clears skin, and weight loss. India is the leading producer of mango (15188000MT) followed by China (4350000MT), Thailand (2600000MT), Indonesia (2131139MT) and Pakistan (1888449MT), respectively. Globally, India contributes 50% of the world's mango production (www.mapsofworld.com). District Chittoor of Andra Pradesh is the leading district in India where maximum production of mangoes. In India, the leading mango producing states are UP AP and Karnataka. Maximum production of mangoes (13876MT), recorded from district Jammu (Figure 28).

**Figure 29:**
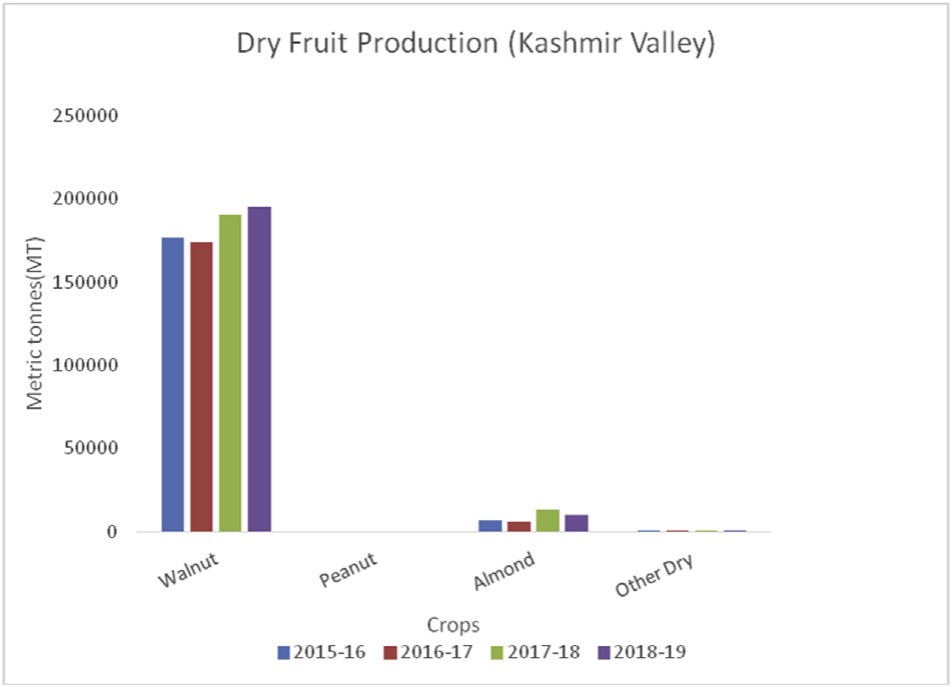
Dry fruit production in metric tons (MT) from 2015-2019 in Kashmir Valley of Union Territory of Jammu & Kashmir.

Litchi (*Litchi chinensis*) (Family: Sapindaceae), is small, delicious with sweet smell and taste, and is extensively grown in China, India, Thailand, Taiwan, and Vietnam. Litchi is rich in water, potassium, carbohydrates, protein, fat, vitamin C, B6, magnesium, iron, and sodium (Shukla et al., 2012). The most important health benefits of litchi are to boost immunity, prevent signs of aging, helps in removing blemishes, reduces sunburns, promote air growth, and make gums strong. China is the global leader of litchi fruits (200000MT), followed by Taiwan (131000), Thailand (10000MT), India (90000) and Madagascar (50000MT) during 2017–18 (www.worldatlas.com). Muzaffarpur district of Bihar is famous for litchi production in India. However, some districts in J&K are producing rich production of these fruits. The maximum production of litchi is from district Jammu (847MT) (Figure 29), followed by Kathua (583MT), Reasi (232MT), Udhampur (163MT) and Samba (138MT).

**Figure 30:**
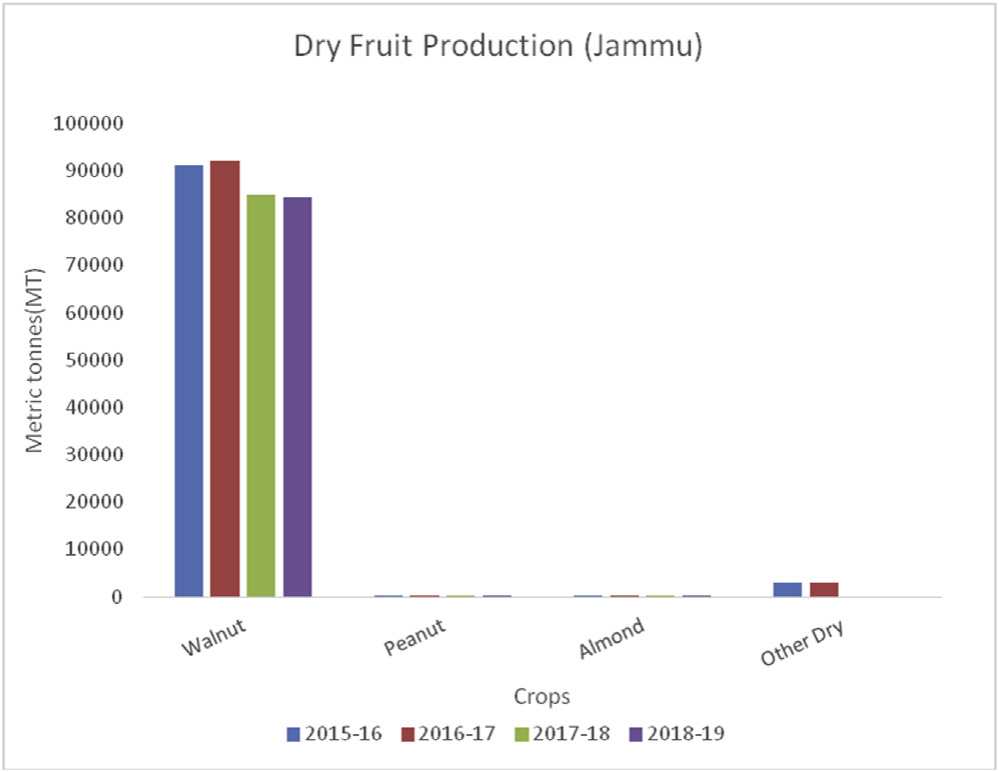
Dry fruit production in metric tons (MT) from 2015-2019 in Jammu region of Union Territory of Jammu & Kashmir.

Ber (*Ziziphus mauritiana*): (Family: Rhamnaceae), is known as poor man's fruit in India. Ber is small, round to oblong, light green to orange-red with a thin glossy peel. They are rich in carbohydrates, fats, proteins, vitamins (B1, B2, and B3), calcium, potassium, iron, and phosphorus (Gao et al., 2013). They are important against insomnia, decrease anxiety, acts as an antioxidant, and boosts immunity. In India, it is widely cultivated throughout the country and in other countries viz., China, Afghanistan, Iran, Russia, Syria, Myanmar, Australia, and the USA. India occupies first place in ber production, with a record production of 5.13 MT from an area of 5000 ha. Madhya Pradesh, Bihar, Uttar Pradesh, and Punjab are the major ber producing states in India. Samba district is the leading producer of ber production (4172MT), followed by Jammu (3335MT), Kathua (1706MT), Rajouri (950MT) and Reasi (100MT) from J& K (Figure 30).

**Figure 31:**
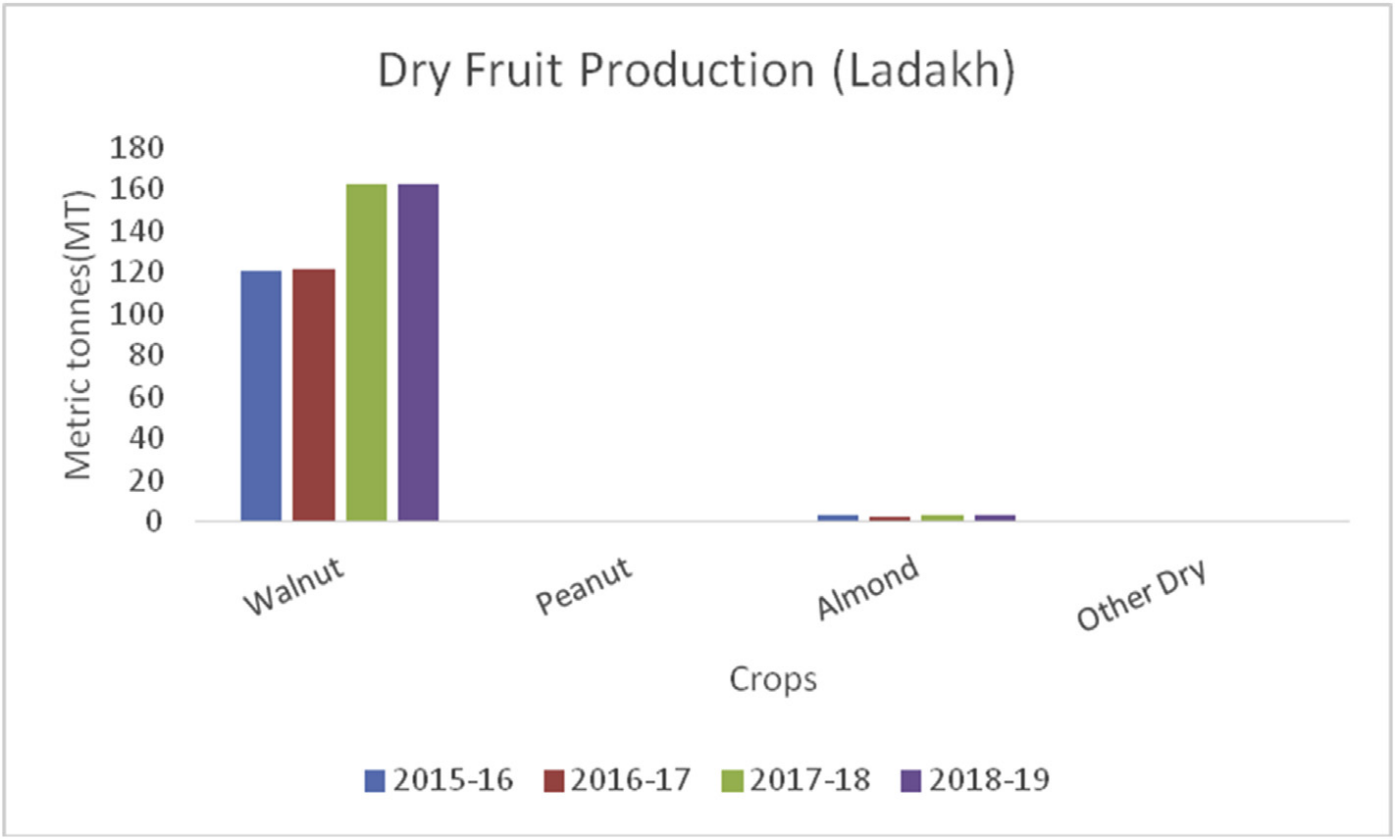
Dry fruit production in metric tons (MT) from 2015-2019 from Union Territory of Ladakh.

Aonla (*Emblica Officinalis*): (Family Euphorbiaceae), is round to prolate in shape and green or yellowish-green in colour, also known as Indian gooseberry or amla. Globally, India is the leading producer of aonla (area & production), followed by Sri Lanka and Cuba. The fruit is a rich source of vitamin C and useful in health as it cures hemorrhages, diarrhea, dysentery, anemia, jaundice, dyspepsia, and cough. Uttar Pradesh and Gujarat are the two states in India, leading in the production of aonla. Jammu district is the leading producer (Figure 31) in aonla production (1506MT), followed by Kathua (1063MT) and Samba (664MT), respectively from the UT of J&K.

**Figure 32:**
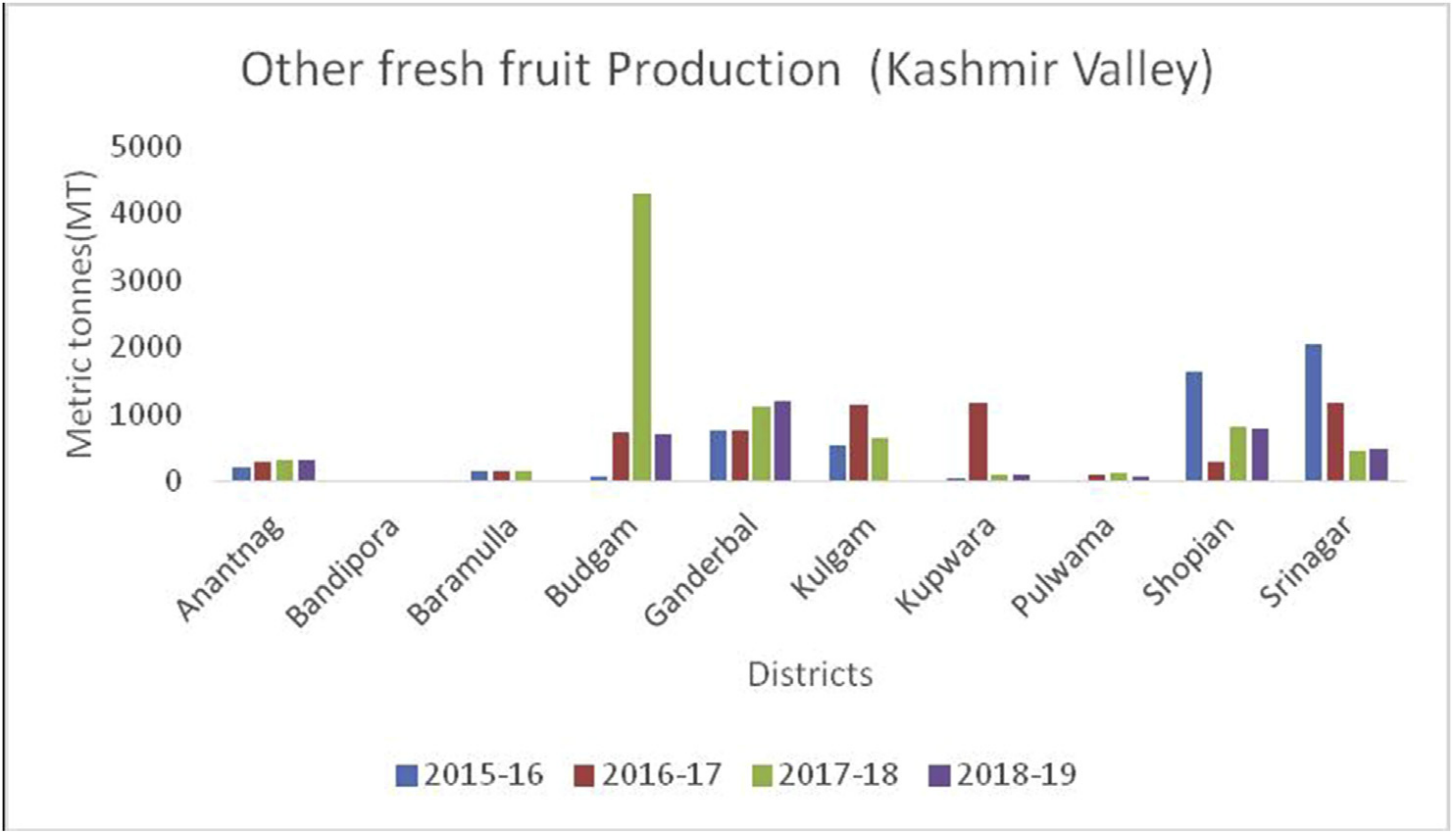
Other fresh fruit production in metric tons (MT) from 2015-2019 in Kashmir Valley of Union Territory of Jammu & Kashmir.

Guava (*Psidium guajava*): (Family Myrtaceae), is spherical or oval, green colour fruit, commonly known as amrood. It possesses anticancer and anti-diabetic properties (Joseph and Priya, 2011), and is useful against anorexia, cerebral ailments, childbirth, chorea, convulsions, epilepsy, nephritis, and jaundice (Kamath et al., 2008). India is a leading producer in guava production (17650000MT), followed by China (436300MT). Thailand is the third-largest producer of 2550600MT, followed by Pakistan (1784300MT) in 2018–2019 (The Daily Records, 2019). Jammu district is leading in guava production (1649MT) followed by Udhampur (1574MT), Samba (1459MT), Kathua (1358MT), Rajouri (1089MT) and Reasi (813MT) from the UT of J&K (Figure 32).

Walnut (*Juglans regia*):(Family Juglandaceae), is an edible seed that originated from the Mediterranean region and central Asia. It is round with green outer pericarp enclosing yellow seed-bearing endocarp with brain-shaped cotyledons which is rich in fat, protein, potassium, sodium, carbohydrate, magnesium, iron, vitamin B6, fiber (6.7%), and antioxidants (Vicente et al., 2009; Sen and Karadeniz, 2015). Walnuts are useful for lowering blood pressure, decreasing inflammation, managing type 2 diabetes, and supporting weight control. China is the leading walnut producer (1000MT) followed by the USA (592.4MT), European Union (113MT), Ukraine (125.9MT), Chile (125MT), Turkey (165MT), and India (35MT) (Sahbaneh, 2020). Globally, China alone contributes 51% of the total walnut production in the world whereas Jammu and Kashmir contribute to around 98% of the total walnut production in India which is followed by HP, AP, and UP.

**Figure 33:**
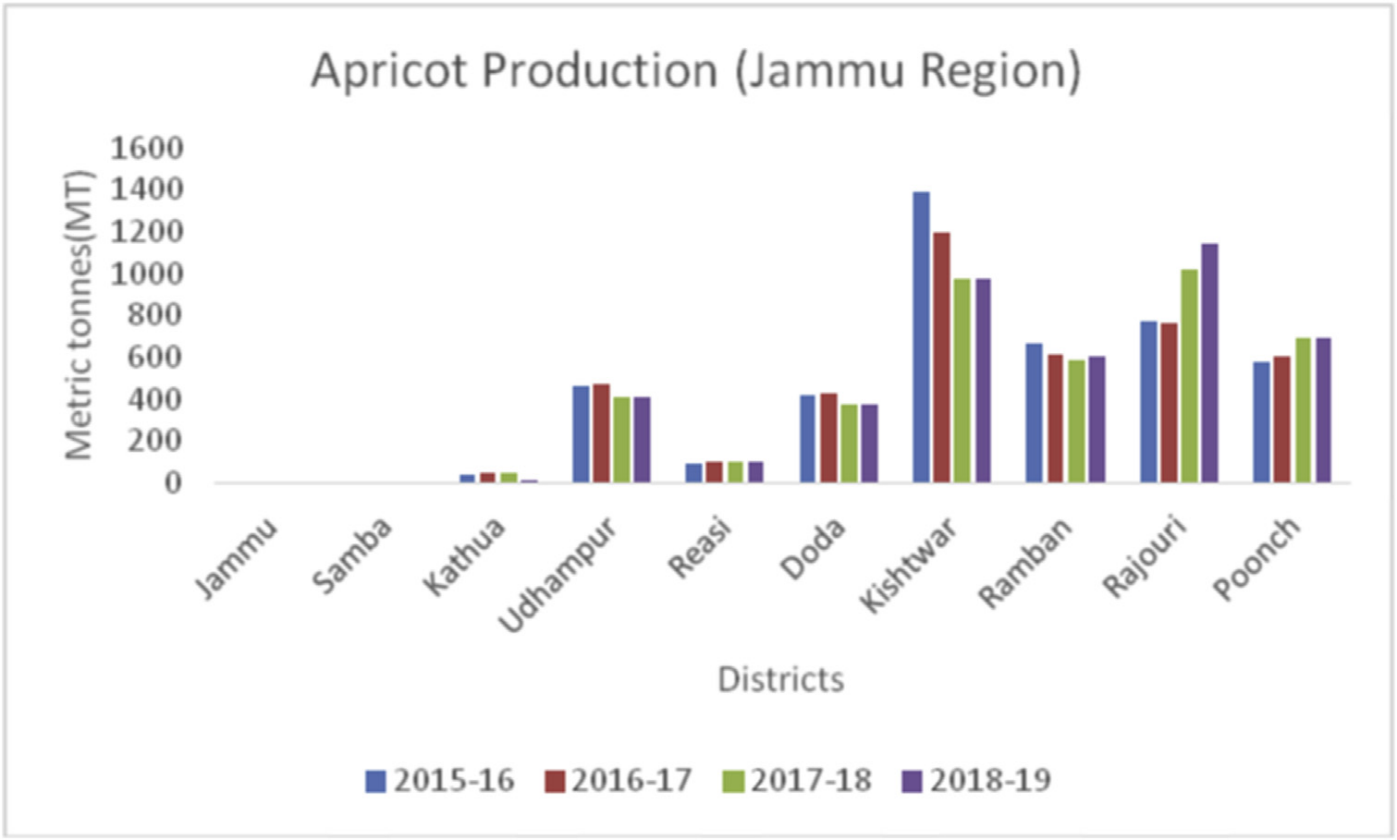
Apricot production in metric tons (MT) from 2015-2019 in Jammu region of Union Territory of Jammu & Kashmir.

**Figure 34:**
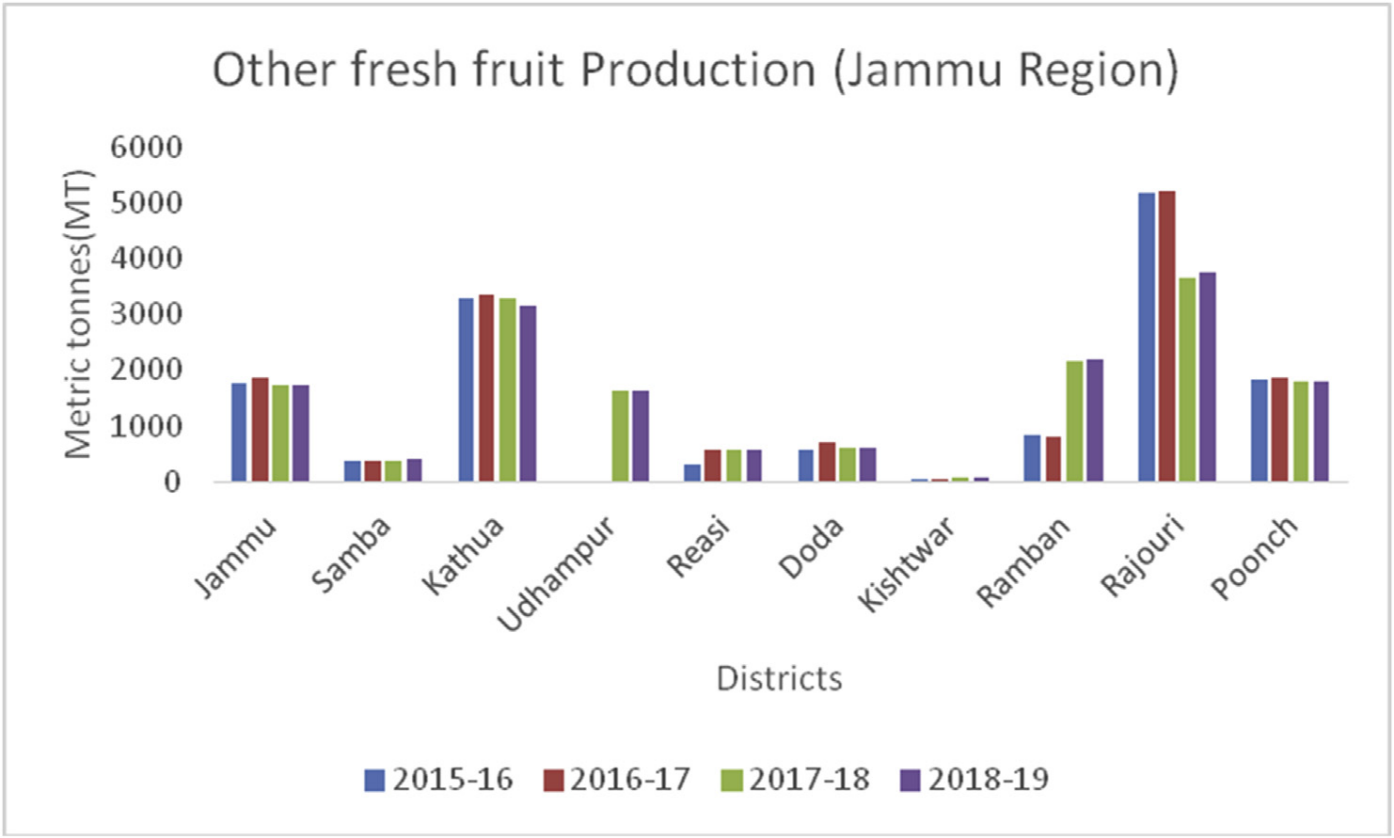
Other fresh fruit production in metric tons (MT) from 2015-2019 in Jammu region of Union Territory of Jammu & Kashmir.

**Figure 35:**
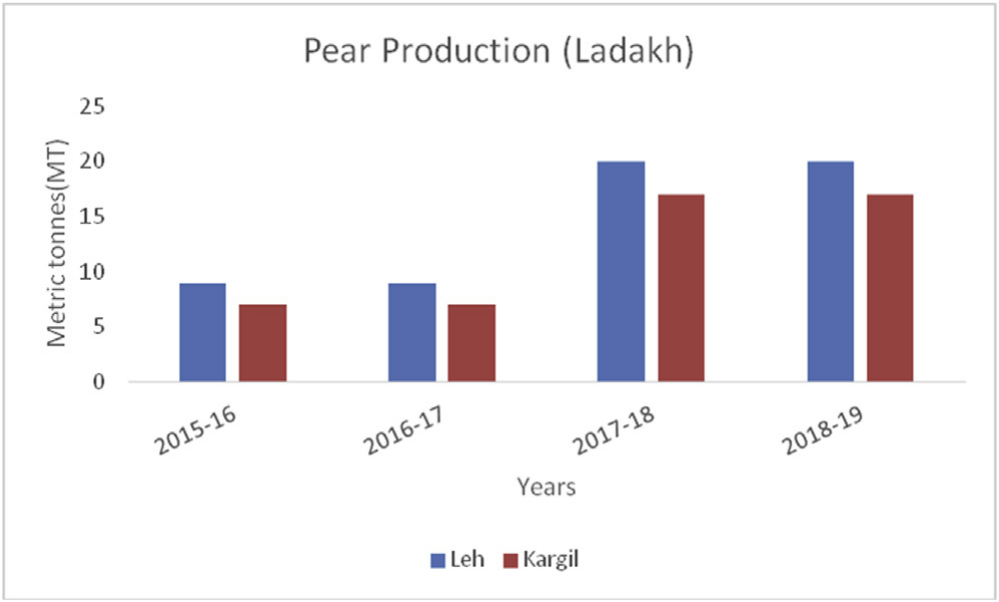
Pear production in metric tons (MT) from 2015–19 from Union Territory of Ladakh.

Peanut (*Arachis hypogaea*):(Family: Fabaceae), is a legume crop and also known as groundnut, grown mainly for its edible seeds. Peanuts are rich in healthful fats (49.66g/100g), water (1.55g/100g), carbohydrates (21.51g/100g), proteins (23.68g/100g), fiber (8.0g/100g), potassium, phosphorous, magnesium, and vitamin B complex (Settaluri et al., 2012). China is the leading producer of peanuts followed by India (15%), the USA, and Nigeria, holding a 40% share of peanut production globally. The main Peanut cultivation states in India are Andhra Pradesh, Gujarat, Tamil Nadu, Karnataka, and Maharashtra. Total area and production under peanuts (Figures 33, 34, and 35) were recorded the last four years from the UT's of Jammu & Kashmir and Ladakh.

Almond (*Prunus dulcis*): (Family: Rosaceae), consumed as edible seeds and are rich in fats, proteins, fiber, calcium, magnesium, phosphorous, potassium, sodium, zinc, copper, manganese, amino acids, and vitamins A, B-complex, E and K (Richardson et al., 2009). Globally, USA is the leading producer of Almonds with an annual production of (1545500MT), followed by Spain (195704MT), Australia (160000MT), Iran (111936MT) and Morocco (101026MT) during 2014–15. In India, almonds are grown in the temperate regions of Jammu & Kashmir, Ladakh, and Himachal Pradesh. Almonds contain lots of healthy fats, fiber, protein, magnesium; calcium and vitamin E. Almonds are beneficial for lowering blood sugar levels, decrease blood pressure and cholesterol, and promote weight loss. Walnut, peanut, almond, and other dry fruit production (Figures 29, 30, and 31) were estimated from both the UT's of Jammu & Kashmir and Ladakh.

## 8 Credit card loan scheme for the farmers and its benefits to the farmers

The Kisan Credit Card (KCC) is a credit scheme introduced by Indian banks on the recommendations of the R.V. GUPTA committee to provide loans to the farmers at a very low interest. The KCC scheme provides financial reforms to rural areas of the country, especially to farmers. The KCC loan is being provided to the customers who are involved with agriculture or other allied activities. The scheme provides short term loans to farmers for purchasing various inputs and other farm needs. The bank officials fix the loan amount based on the area under farming, type of crop under cultivation, history of the farmer, seeds, fertilizers, expenses related to crop production, protection, and other factors. Mostly the farmers seek State Bank of India credit card loans compared to other banks as the interest rate in this bank is being charged from 2 % per annum and a loan up to 3.0 lakhs.

The loan is being provided to the farmers at cheaper rates. Besides, the KCC cardholders are covered under the crop insurance scheme as well as personal accident insurance. The validity of the scheme is for five years with an option to continue it up to three more years. Two types of credit are being provided to the farmers viz; cash and term credit. On timely payment, the interest rate of 4 % is being charged to the farmers and the card limit increased from Rs. 1.60 to Rs 3 lakh. The KCC scheme spread out rapidly, as banks are not demanding security for the loan amount up to Rs. 1.60 Lakhs from the farmers. The government-run banks of India have introduced a waiver of about 3 % if the farmer repays the loan timely. After the due date, such banks will accelerate the interest rate and charge on the loan amount with an interest rate of 7%. The KCC also provides interest to loan accounts of farmers on their savings.

## 9 Conclusion

Fruits and vegetables are the basic need and important sources of generating income for local farmers. The horticulture sector will create sufficient job opportunities and improve the dietary requirements in the form of micronutrients and vitamins. There is a great need to develop a protocol for the organic production of fresh fruits in Jammu & Kashmir and Ladakh and also for their marketing. The future of the nation depends upon the skilled, spirited, and hopeful youth with a positive outlook. So maximum youth living in rural areas may be involved through various Government schemes to curb unemployment. Subsequently, Govt. of India should operate various centrally sponsored schemes in these newly created Union territories so that the growers may get an opportunity to grow high-quality fruits with maximum output and with small inputs. Such schemes in the horticulture sector may lead to the growers more capable, productive, and resilient. Some missions in foreign countries like Eco Apples, Red Tomatoes, etc should also be flourished in these territories to connect farmers and consumers through marketing, trade, and education. Indian govt. should have a definite goal in creating a niche market for all dominant fresh fruits. The less dominant, thin-skinned fresh fruits (Cherry) will fetch the best prices and access to high-quality markets if such niche markets may be created for the benefit of growers. Growers are claiming the shortage of advanced and modern facilities due to the dearth of manual labour during the peak seasons (harvesting and pruning and training) of their fruit crops. Although various inputs, like fertilizers and pesticides, are made available by the government through various national and international companies, still there are gaps to improve their livelihood security for their better living. The high-quality planting material for the growers, urban population, and backyard growers are not fully ensured by the government at the grass-root level. The central sector plan scheme called Crop Estimation Survey of Fruits and Vegetables (CES-F&V) should be implemented in Jammu & Kashmir and Ladakh like other states, to benefit the farming community. Around 25 fruits are cultivated in J&K and the Plant protection advisory has been made available for few major fruit crops viz; apple, pear, cherry, and walnut against various insect pests and diseases for their management, developed by the Department of Horticulture, J&K. Monitoring and setting the biofix for various pests/diseases are not being practiced annually based on degree-day models using spore and pheromone traps (Hussain et al., 2015). They are unable to predict the population of boring insects (Stem/fruit borers) and diseases on apples in advance. The use of pheromone dispensers is not gaining momentum in India for the management of various insect pests as compared to western/developed nations. The pheromone dispenser technology is not being registered against any insect pest in India. To boost organic production, pheromone dispenser technology will cut down pesticide usage and environmental pollution. The will and comprehensive deliberations are needed to reduce the pesticide usage and strict policy of the Government of India to replace these toxic insecticides from the markets of India. There are various options already available for managing various insect pests and diseases on fresh fruits and diseases to increase the yield of these crops. Subsequently, the pesticide residues in fresh and dry fruits could not fulfill the requirements for fruit trade in global markets according to WTO agreements. Fresh fruit growers of India are in distress not because they are unable to produce but unable to sell to global markets due to the spray of highly toxic insecticides. Western and other Asian countries already banned Chlorpyriphos, the highly toxic insecticide (Bonvoisin et al., 2020). In India, the chlorpyriphos insecticide is available to growers at cheaper rates and also made available to them by the enforcement agencies of India. The other commercial fruits cultivated in J& K and Ladakh are ignored due to financial constraints. The information on modern scientific methods (production and protection technologies) adopted by the western developed nations regarding their cultivation is lacking. The scientific interventions in plant sciences already developed by the western countries for both fresh and dry fruits are not being followed as compared to medical sciences by the government of India. Though such technologies can be easily shared and adopted. The benefit of online resources for the production and protection technologies adopted by the western countries for all these crops is easily available on the internet to mitigate the problems of growers. Crop diversification is a promising strategy in the horticulture sector which is very convenient and economical to boost the economy of local growers. The roads are not properly maintained and also not connected to fruit orchards. Hence the hyper transportation cost makes the fruits very expensive in terms of competing with fruits of other states of the Indian union. These fresh fruits are highly perishable and could not find a market at the proper time and have to perish on the way due to scarcity of refrigerated trucks.

Jammu & Kashmir has witnessed a tremendous increase in horticulture production over the last decade. Significant progress has been made in area expansion resulted in higher production. The total production of horticulture fruits is maximum in district Baramulla (19.61%) followed by Kupwara (14%), Shopian (13.14%), and Kulgam (10.40%). From the last decade, it has been observed that only some districts are leading in horticulture production in J&K. Although, variable environmental conditions, topography, soil fertility, water availability, poor production, and protection strategies, and variable geographic factors are the ample reasons for the poor production of fresh fruits in India (Lone and Sen, 2014). Our results revealed that every district has its potential for horticulture production especially the type of soils, the fertility of the land, and other geographical factors. In this regard, the Government of India (GoI) should explore new techniques and technologies to consult experts to maintain and sustain the growth of fresh fruits. Accurate data of the area and production of both fresh and dry fruits should be maintained properly through concerned agencies to predict the area and production of these fruit crops. The various technologies developed and demonstrated by SKUAST-K for the growth and development of horticulture may be implemented and evaluated under area-wide management Programmes. Recent scientific interventions are to be adopted to generate more income and employment opportunities to save the horticulture industry. The introduction of high-density plantations (HDP) of temperate fruits (Apple, pear, cherry, apricot, walnuts) revolutionized the horticulture industry of J&K but still, there is a need to motivate the commercial/marginal fruit growers to convert their traditional orchards under high-density plantations to boom the temperate horticulture industry of India. Due to temperate climatic conditions, the monopoly of cultivating specific fruit crops than the other states of the Indian union will boost horticulture production and livelihood security of the growers of J&K and Ladakh. Although, market intervention scheme is a good initiative proper marketing facilities should be provided to the growers all the time for all kinds of fruits. The government of India is giving a lot of emphasis on the horticulture and agriculture sectors for increasing the production and productivity of agricultural and horticulture commodities to boost the Indian economy and trade. Many new initiatives like the National Horticulture Mission (NHM), Technology Mission for Integrated Development of Horticulture in the North Eastern States (TMNE), National Food Security Mission (NFSM), Rashtriya Krishi Vikash Yojna (RKVY), National Project on Organic Farming (NPOF), Krishi Vigyan Kendra's (KVK's), National Project on Management of Soil Health and Fertility (NPMSHF) for the development of horticulture industry. The success of these schemes will lead to a significant upsurge in agriculture/horticulture production if implemented properly. There is no separate production and management capsule available to growers who are raising fruit crops in irrigated and karew areas (Irfan et al., 2019). Nowadays, the growers of Karewa areas are facing a lot of difficulties such as scarcity of road connectivity, water problems due to changing climatic conditions, difficulties in converting their traditional orchards into high-density plantations. The high-density plantations require more water as they have shallow rootstocks and mostly the plants fail to grow in Karewa belts. The policy of establishing the deeply rooted rootstocks in the karewa's may be available to the growers. Besides, the introduction of new early and mid-early maturing varieties will ensure the early introduction of these fruits in such niche markets. Planting varieties of deep rootstock in Karewa belts will ensure higher yields. From the last few years, snowfall has witnessed early in November that leads to a sudden increase of apple scab as orchard sanitation, punning and training and other practices could not be performed timely. Mostly, all the intercultural practices in both the UT's of J&K and Ladakh are still being carried out manually. To the best of our knowledge, growers of Ladakh are very poor in orchard management, and the killing of insect pests in some pockets of the Leh region is not being practiced because of social taboos. The dissemination of pheromone baited traps/dispenser technology in Nurla village revealed promising results to motivate the grower to adopt this technology. The ban on all fresh fruits from Ladakh is still considered as a good rationale to protect the apple bowl of India from the infestation of codling moth. Kissan credit card (KCC) scheme for the farmers is a good initiative operated by the government of India, to boost the economy of growers. This scheme was launched by NABARD to meet out the comprehensive credit requirements of the agriculture sector by giving financial support to growers. But its rate of interest should be reduced to 2% that is currently 8% and 4% and every grower and crop must be brought under various insurance schemes. The mission for Integrated Development of Horticulture is a centrally sponsored scheme for the holistic growth of the horticulture sector covering fruits, vegetables, root and tuber crops, mushrooms, spices, flowers, aromatic plants, cashew, cocoa, coconut, and bamboo. Although, this scheme is good, and the share of the central Govt. is not equal in every state of India. The share of 100% by the central government of India must be provided to the territories of Jammu & Kashmir and Ladakh. Govt. of India must organize some programs and events to create awareness to local growers about various central schemes in horticulture and agriculture for their implementation at the grass-root level. Factories, research institutes and other infrastructure related to the horticulture sector must be established in Jammu & Kashmir as well as in Ladakh for the benefit of growers and to increase employment in this sector. The suggestions put forward in this manuscript will boost the production of both fresh and dry fruits in Jammu & Kashmir and Ladakh. The monopoly in the production of specific fruit crops in these territories will ensure quality fruit production and productivity and will be a major contribution to trickle down the imports of these fresh fruits in India from across the world.

## Declarations

### Author contribution statement

Rayees Ahmad Bhat, Barkat Hussain: Conceived and designed the experiments; Wrote the paper.

Tariq Ahmad: Analyzed and interpreted the data.

### Funding statement

This work was supported by 10.13039/501100001407DBT (102/IFD/SAN/4671/2017-2018 Dated: 13-03-2017).

### Data availability statement

Data included in article/supplementary material/referenced in article.

### Declaration of interests statement

The authors declare no conflict of interest.

### Additional information

No additional information is available for this paper.

## References

[bib1] Angmo P., Angmo S., Upadhyay S.S., Targais K., Kumar B., Stobdan T. (2017). Apricots (*Prunus armeniaca* L.,) of trans-Himalayan Ladakh: potential candidate for fruit quality breeding programs. Sci. Hortic..

[bib4] Bondonno N.P., Bondonno C.P., Ward N.C., Hodgson J.M., Croft K.D. (2017). The cardiovascular health benefits of apples: whole fruit v/s isolated compounds. Trends Food Sci. Technol..

[bib5] Bonvoisin T., Utyasheva L., Knipe D., Gunnell D., Eddleston M. (2020). Suicide by pesticide poisoning in India: a review of pesticide regulations and their impact on suicide trends. BMC Publ. Health.

[bib6] Daliakopoulos I.N., Tsanis I.K., Koutroulis A., Kourgialas N.N., Varouchakis A.E., Karatzas G.P., Ritsema C.J. (2016). The threat of soil salinity: a European scale review. Sci. Total Environ..

[bib8] Fatima T., Bashir O., Gani G., Bhat T.A., Jan N. (2018). Nutritional and health benefits of apricots. Int. J. Unani Integr. Med..

[bib10] Gao Q.H., Wu C.S., Wang M. (2013). The jujube (Ziziphus jujuba Mill.) fruit: a review of current knowledge of fruit composition and health benefits. J. Agric. Food Chem..

[bib15] Goyal A. (2020). Post Covid-19: Recovering and Sustaining India's Growth.

[bib18] Hindustan Times (May, 2019). Leading National Newspaper Publishing Date.

[bib19] Hussain M. (2000). Systematic Geography of Jammu and Kashmir.

[bib20] Hussain B., Ahmad B., Bilal S. (2015). Monitoring and mass trapping of the codling moth, *Cydia pomonella*, by the use of pheromone baited traps in Kargil, Ladakh, India. Int. J. Fruit Sci..

[bib21] Imran M., Rauf A., Imran A., Nadeem M., Ahmad Z., Atif M., Awais M., Sami M., Fatima Z., Waqar B.A. (2017). Health benefits of grapes polyphenols. J. Environ. Agric. Sci..

[bib22] Irfan M., Qadir A., Ali H., Jamil N., Ahmad S.R. (2019). Vulnerability of environmental resources in Indus Basin after the development of irrigation system. Irrigation-Water Productivity and Operation, Sustainability and Climate Change.

[bib24] Joseph B., Priya M. (2011). Review on nutritional, medicinal and pharmacological properties of guava (Psidium guajava Linn.). Int. J. Pharma Bio Sci..

[bib25] Kamath J.V., Rahul N., Kumar C.K.A., Lakshmi S.M. (2008). *Psidium guajava* L: a review. Int. J. Green Pharm..

[bib26] Kelly D.S., Adkins Y., Laugero K.D. (2018). A review of the health benefits of cherries. Nutrients.

[bib28] Kumar P., Sethi S., Sharma R.R., Singh S. (2018). Nutritional characterization of apple as a function of genotype. J. Food Sci. Technol..

[bib50] Lone A.L., Sen V. (2014). Horticulture sector in Jammu and Kashmir, Economy. Eur. Acad. J..

[bib30] Misachi J. (2017). The World's Top Citrus Producing Countries. https://www.Worldatlas.com/2017.

[bib31] Morris J.R., Cawthon D.L., Fleming J.W. (1980). Effects of high rates of potassium fertilization on raw product quality and changes in pH and acidity during storage of Concord grape juice. Am. J. Enol. Vitic..

[bib32] Moyer R.A., Hummer K.E., Finn C.E., Frei B., Wrolstad R.E. (2002). Anthocyanins, phenolics, and antioxidant capacity in diverse small fruits: Vaccinium, Rubus, and Ribes. J. Agric. Food Chem..

[bib33] Muzaffar S., Mir M.A., Dar Z.A., Dar N., Khan K.H. (2018). Effect of soil constituents on the chemical characteristics of Saffron. Parma Innov..

[bib34] Nile S.H., Park S.W. (2014). Edible berries: bioactive components and their effect on human health. Nutrition.

[bib35] Pantelidis G., Vasilakakis M., Manganaris G., Diamantadis G. (2007). Antioxidant capacity, phenol, anthocyanin and ascorbic acid contents in raspberries, blackberries, red currants, gooseberries and Cornelian cherries. Food Chem..

[bib36] Pillai R.G. (2020). Antiproliferative actions of Ziziphus jujube fruit extract is mediated through alterations in Bcl2-Bax ratio and through the activation of caspases. Chem. Biol. Lett..

[bib37] Rehman M.U., Hussain B., Mir M.M., Angmo T., Parray E., Zubair M. (2020). Low productivity of fruits, its implications and combating strategies in cold arid eco-region of Ladakh (J&K). Curr. J. Appl. Sci. Technol..

[bib38] Reiland H., Slavin J. (2015). Systematic review of pears and health. Food Nutr..

[bib39] Richardson D.P., Astrup A., Cocaul A., Ellis P. (2009). The nutritional and health benefits of almonds: a healthy food choice. Food Sci. Technol. Bull. Funct. Foods.

[bib40] Romera F.J., Alcántara E., De La Guardia M.D. (1991). Characterization of the tolerance to iron chlorosis in different peach rootstocks grown in nutrient solution. Iron Nutrition and Interactions in Plants.

[bib41] Sahbandeh M. (2020). Major Apple Producing Countries Worldwide 2019/2020. https://www.statista.com.

[bib51] Sangral C. (2015). District-wise production of fresh and dry fruits in Jammu and Kashmir. Int. J. Econ. Bus. Rev..

[bib42] Sen M., Karadeniz T. (2015). The nutritional value of walnut. J. Hyg. Eng. Design.

[bib43] Settaluri V.S., Kandala C.V.K., Puppala N., Sundaram J. (2012). Peanuts and their nutritional aspects- A review. Food Nutr. Sci..

[bib44] Shukla R.K., Painuly D., Porval A., Shukla A. (2012). Proximate analysis, nutritive value, total phenolics content and antioxidant activity of *Litchi chinensis* sonn. Nat. Products.

[bib45] Sing H.V. (2013). Soil carbon sequestration and rhizospheric microbial population in apricot orchards following plastic film mulching under cold arid region. Int. J. Hortic..

[bib46] The Daily Records (2019). Top ten largest guava producing countries in the world. http://www.thedailyrecords.com.

[bib47] Turner T., Burri B.J. (2013). Potential nutritional benefits of current citrus consumption. Agriculture.

[bib48] USDA (2020). Agricultural Research Service. https://www.ars.usda.gov.

[bib49] Vicente A.R., Manganaris G.A., Sozzi G.O., Crisosto G.H. (2009). Nutritional quality of fruits and vegetables. Postharvest Handling, a System Approach.

